# Redefining phenotypic intratumor heterogeneity of pancreatic ductal adenocarcinoma: a bottom‐up approach

**DOI:** 10.1002/path.6398

**Published:** 2025-02-11

**Authors:** Marc Hilmi, Flore Delecourt, Jérôme Raffenne, Taib Bourega, Nelson Dusetti, Juan Iovanna, Yuna Blum, Magali Richard, Cindy Neuzillet, Anne Couvelard, Matthieu Tihy, Louis de Mestier, Vinciane Rebours, Rémy Nicolle, Jérôme Cros

**Affiliations:** ^1^ Molecular Oncology Team, UMR 144 Institut Curie Paris France; ^2^ Medical Oncology Department Institut Curie Saint‐Cloud France; ^3^ Pathology Department, APHP Nord, FHU MOSAIC Beaujon Hospital Clichy France; ^4^ JR‐Analytics Pompignac France; ^5^ Centre de Recherche sur l'Inflammation (CRI), INSERM, U1149, CNRS, ERL 8252 Université Paris Cité Paris France; ^6^ Centre de Recherche en Cancérologie de Marseille (CRCM), INSERM U1068, CNRS UMR 7258, Parc Scientifique et Technologique de Luminy Aix‐Marseille Université and Institut Paoli‐Calmettes Marseille France; ^7^ Univ Rennes, CNRS, INSERM, IGDR (Institut de Génétique et Développement de Rennes) – UMR 6290, ERL U1305 Rennes France; ^8^ Université Grenoble Alpes, CNRS, UMR 5525, VetAgro Sup, Grenoble INP, TIMC Grenoble France; ^9^ Pathology Department, APHP Nord Bichat Hospital Paris France; ^10^ Department of Pancreatology and Digestive Oncology, APHP Nord, FHU MOSAIC Beaujon Hospital Clichy France

**Keywords:** pancreatic ductal adenocarcinoma, image analysis, molecular subtypes, tumoral heterogeneity

## Abstract

Pancreatic ductal adenocarcinoma (PDAC) tumor interpatient heterogeneity has been well described with two major prognostic subtypes (classical and basal‐like). An important intrapatient heterogeneity has been reported but has not yet been studied extensively due to the lack of standardized, reproducible, and easily accessible high‐throughput methods. We built an immunohistochemical (IHC) tool capable of differentiating RNA‐defined classical and basal‐like tumors by selecting relevant antibodies using a multistep process. The successive stages of (i) an *in silico* selection from a literature review and a bulk transcriptome analysis of 309 PDACs, (ii) a tumor‐specific selection from 30 patient‐derived xenografts and single‐cell data, followed by (iii) the validation on tissue microarrays in 50 PDAC were conducted. We used our final IHC panel on two independent cohorts of resected PDAC (*n* = 95, whole‐slide, *n* = 148, tissue microarrays) for external validation. After digitization and registration of pathology slides, we performed a tile‐based analysis in tumor areas to identify relevant marker combinations. Sequential marker selection led to the following panel: GATA6, CLDN18, TFF1, MUC16, S100A2, KRT17, PanBasal. Four different phenotypes were identified: one classical, one intermediate (KRT17+), and two basal‐like (MUC16+ versus S100A2+) with specific biological properties. The presence of a minor basal contingent drastically reduced overall survival [hazard ratio (HR) = 1.90, *p* = 0.03], even in classical predominant PDACs. Analysis of preneoplastic lesions suggested that pancreatic carcinogenesis might follow a progressive evolution from classical toward a basal through an early intermediate phenotype. In conclusion, our IHC panel redefined and easily assessed the high degree of intra‐ and intertumoral heterogeneity of PDAC. © 2025 The Author(s). *The Journal of Pathology* published by John Wiley & Sons Ltd on behalf of The Pathological Society of Great Britain and Ireland.

## Introduction

Pancreatic ductal adenocarcinoma (PDAC) has one of the poorest prognoses due to the fact that its diagnosis is often made at a late stage and it has low sensitivity to therapies. Two major PDAC transcriptomic subtypes have been described: a classical subtype, which expresses epithelial differentiation genes, with a better prognosis than the basal‐like subtype, which expresses epithelial‐mesenchymal transition genes [1]. Yet recent studies questioned the existence of such a blunt dichotomy at the tumor cell level by highlighting frequently misclassified or hybrid/mixed tumors [2, 3, 4].

PDAC displays not only interpatient but also intrapatient heterogeneity. From a histological standpoint, PDAC is heterogeneous as different histological architectures have been described and can coexist within the same tumor introducing the concept of intratumoral epithelial heterogeneity [5, 6]. The squamous/non‐gland‐forming morphology is associated with the basal‐like subtype, whereas the glandular/gland‐forming morphology is associated with the classical subtype [6, 7]. However, within the classical transcriptomic subgroup, there seem to be tumors or parts of tumors that do not form glands and have a worse prognosis than purely glandular tumors [2, 6]. At the RNA level, bulk transcriptomic analyses are costly and difficult to implement in clinical practice and provide a global reflection of tumors, without being able to reliably highlight any heterogeneity. Consequently, a continuous molecular gradient was proposed to better recapitulate the histological groups, making it possible to order the tumors based on their ‘classicness or basalness’ but with no information on intratumor heterogeneity [8]. Several single‐cell studies helped to decipher intratumor heterogeneity and suggested that there was a co‐occurrence of the classical and basal‐like subtypes within the same PDAC (hybrid or mixed tumors) [3, 7, 9, 10, 11]. Moreover, Williams *et al* [10] showed that cells co‐expressing classical and basal markers were present in over 90% of PDACs and could constitute an intermediate phenotype. However, these approaches have been applied to a small number of patients, restricting the generalizability of their conclusions. Our team recently showed the possibility of classifying cases into subtypes based on histological features from hematoxylin and eosin (H&E) staining through a deep learning model [2]. In addition to the hybrid tumors combining classical and basal components, intermediate tumors for which most tumor cells could not be morphologically clearly assigned to either of the two classical and basal subtypes were revealed. Nevertheless, the morphological approach is time consuming to fully assess the extent of intratumor heterogeneity and requires expertise in pancreatic pathology. Multiarea microdissection constitutes an interesting approach to exploring intratumor heterogeneity but cannot be used to evaluate heterogeneity within a single gland. Overall, the definition and estimation of intratumor heterogeneity on a large scale is currently not feasible due to the lack of a standardized and easily accessible method.

The strength of immunohistochemistry (IHC), compared to transcriptomic or H&E‐stained image analysis of tumors, lies in the fact that this technique allows, in an easy and cost‐effective way, a fine analysis of each tumor cell conserving its spatial localization. Our objective was to build a robust IHC panel reflecting molecular subtypes in order to explore PDAC intratumor heterogeneity.

## Methods

### Patient samples

The *in silico* marker selection was performed on a retrospective multicenter cohort, including 309 resected PDACs in four university hospitals as described previously (Cohort 1) [12]. In brief, exclusion criteria were neoadjuvant chemotherapy or radiochemotherapy, macroscopically incomplete surgical tumor resection (R2), and a histologic diagnosis other than adenocarcinoma. The bulk transcriptome of these tumors was obtained using microarrays (Affymetrix HGU219, Affymetrix Pte Ltd, Singapore), as described previously [12]. From this cohort, 13 clear classical and eight clear basal‐like tumors from the transcriptomic point of view were selected to construct a tissue microarray (TMA) with four spots of 0.6 mm/case.

The previously published transcriptomes of 30 patient‐derived xenografts, for which the human tumor compartment was separated from the mouse stromal compartment and tumor subtyping was performed on the tumor compartment, was obtained from the PaCaOmics Clinical Trial (NCT01692873) [13].

The human validation of the IHC markers on whole slides was performed on a cohort of 50 PDAC cases from Beaujon Hospital with the same inclusion criteria as the cohort described above. The bulk transcriptome of these cases was obtained after macrodissection of the whole tumor area on the same block as the one used for the IHC and obtained from formalin‐fixed paraffin‐embedded (FFPE) sections using a high‐purity FFPE RNA isolation kit (Roche, Basel, Switzerland) following the manufacturer's protocol. Library preparation was performed using QuantSeq 3’‐mRNA‐Seq REV (Lexogen GmbH, Vienna, Austria) with an input of 150 ng of total FFPE RNA. To assess the intratumor heterogeneity on a larger scale, the final IHC panel was performed on an additional cohort of 45 cases with the same inclusion criteria, leading to a total cohort of 95 patients for the tile‐based analysis (Cohort 2).

In addition, a cohort of 148 resected PADCs already described (Cohort 3) was used to construct a TMA with four spots of 0.6 mm/case and to validate the prognostic impact of the IHC panel [2]. The techniques performed on the three cohorts of patients with resected PDACs, and their aims are presented in supplementary material, Figure S1.

Finally, a treatment‐naïve cohort of 53 paired samples (primary PDAC and liver metastases) obtained by endoscopic ultrasound fine‐needle aspiration was used to perform RNA sequencing (RNA‐seq) (RNAeasy FFPE kit, Qiagen, Venlo, The Netherlands) (Cohort 4) [14].

This study was performed in accordance with the Declaration of Helsinki (1975), as revised in 1983, and was approved by the ethics review board. Patient informed consent forms were collected and registered in a central database.

### Immunohistochemistry

All IHC staining was performed using a routine automated platform (Ventana Benchmark Ultra, Roche diagnostic, Tucson, AZ, USA). The details of each antibody used are listed in supplementary material, Table S1. For the spatial analysis, IHC staining was performed on five serial slides: three slides with a duplex IHC to limit the number of slides (GATA6/S100A2 – PanBs/TTF1 – KRT17/CLDN18) and two slides with a single marker MUC16 and Pan Cytokeratin. The stains were assessed using H‐scores, defined as the staining intensity (0 = null, 1 = weak, 2 = moderate, 3 = strong) multiplied by the percentage of positive tumor cells (ranging from 0% to 100%) leading to a score ranging from 0 to 300. No spot without tumor cells was scored.

### Tumor differentiation

The grading was performed by expert pathologists (AC and JC) following the World Health Organization (WHO) guidelines, except for the mitosis count, which is not part of the American or the French pathology guidelines for PDAC [15]. Tumors were therefore presented as well, moderately, and poorly differentiated rather than in accordance with the WHO's G1–G4.

### Image analysis

Registration of digitized whole‐slide images of serial histology sections was performed using VALIS software [16]. Manual annotations on H&E‐stained images were made by an expert pathologist (JC), and virtual tile cutting and pixel detection were conducted using QuPath version 0.4.3 [17]. The algorithm for pixel detection was based on a pixel classifier and was trained on representative pictures. Filtration of tiles was based on their size and content of PANCK and performed using R software version 4.3.0.

### Single‐cell RNA‐seq

Single‐cell RNA‐seq raw data from Peng *et al* [18] were retrieved. Using the Seurat V5 R package [19], data preprocessing functions were run with default parameters. Low‐quality cells (<200 genes/cell, <3 cells/gene, and >5% mitochondrial genes) were excluded. Highly variable genes were identified and used to perform principal component analysis (PCA). Significant principal components 1 to 20 were determined using JackStraw. We identified clusters of cells by a shared nearest neighbor (SNN) modularity optimization‐based clustering algorithm with Seurat's implementation at a resolution of 0.5. Cell clusters were projected onto t‐distributed stochastic neighbor embedding (t‐SNE) analysis using previously computed principal components. Cell types were retrieved from the original publication.

### Statistical analyses

Tile clustering was performed on logged values of pixel proportions by the method of k‐means using the first components of PCA counting for more than 5% of the variability. The optimal number of clusters was based on the silhouette method. Cluster expression was classified according to their presence (>1%, as below can be related to detection artifacts) and predominance (>50%, as the major cluster). We used the Kaplan–Meier method to provide median survival times. The log‐rank test was used to evaluate statistical significance. We used Cox proportional hazards regression to evaluate associations between tumor clusters and overall survival (OS). Potential prognostic factors included in the models were age >65 years, resection margins, vascular embolism, lymph node, and vascular and perineural invasion. Pathway analyses were performed with gene set enrichment analysis (GSEA) using the DESeq2 package. All statistical analyses were performed with R software version 4.3.0, and statistical significance was set at a two‐tailed *p* value of <0.05.

## Results

### Development of IHC panel to distinguish basal‐like and classical subtypes

**Figure 1:**
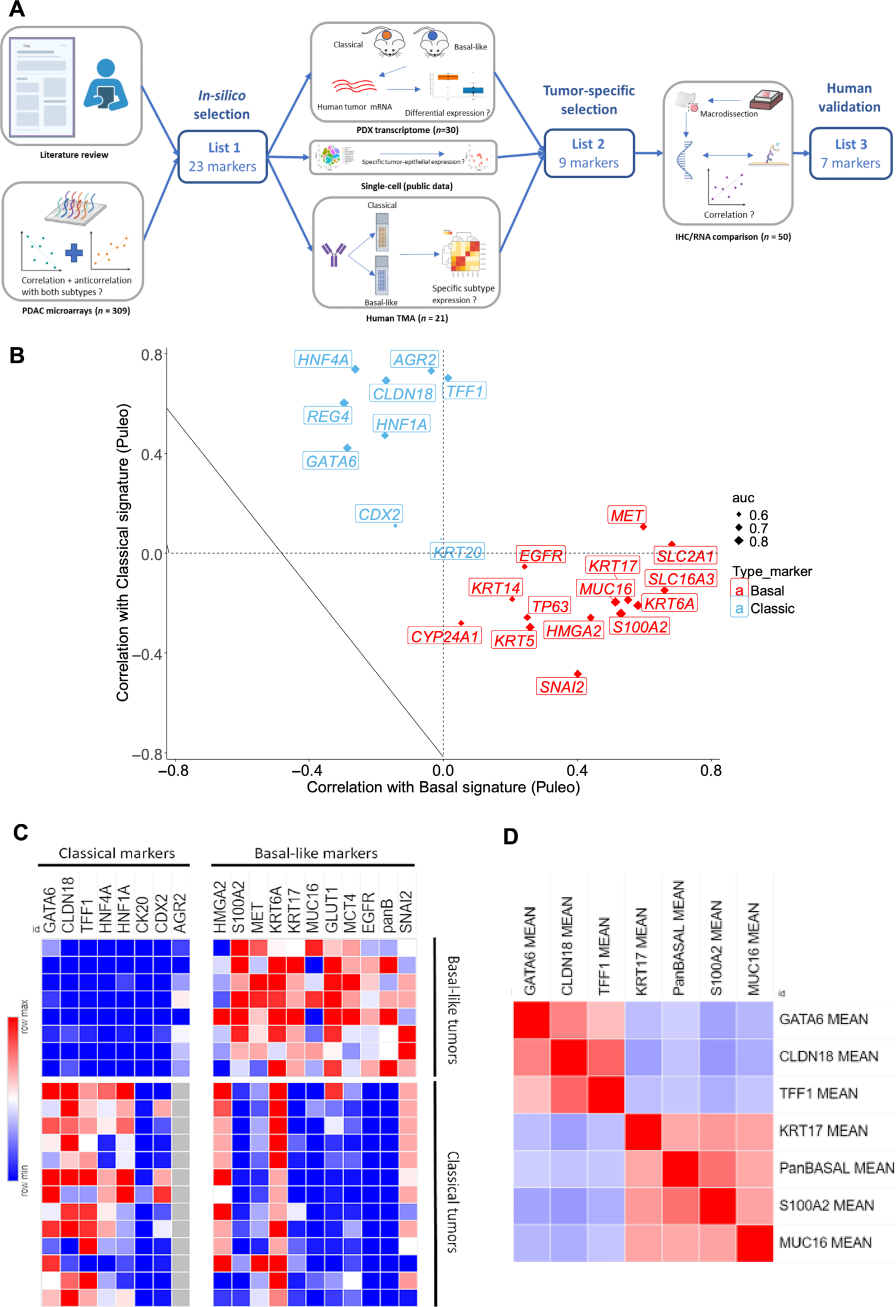
Marker selection to determine molecular subtype. (A) Multistep process to identify IHC marker surrogate of classical and basal‐like subtypes. IHC, immunohistochemistry; PDAC, pancreatic ductal adenocarcinoma; PDX, patient‐derived xenografts; TMA, tissue microarray. Created with Biorender.com. (B) Scatterplot showing correlation of gene expressions according to classical and basal signatures from Puleo *et al* (Cohort 1). Size of points is modulated by value of AUC predicting Purist signature. Basal markers are in red, classical markers in blue. (C) Heatmap representing IHC expression of tested markers in set of 13 classical and eight basal‐like pancreatic ductal adenocarcinomas from Cohort 1. H‐score: 0 (blue) to 300 (red). (D) Correlation matrix of the seven validated IHC markers in 50 human PDACs from Cohort 2 (red = strong positive correlation, blue = strong negative correlation).

To build a robust IHC tool capable of differentiating classical and basal‐like tumor cells, we selected the relevant markers using a multistep process (Figure 1A). IHC markers should (i) be highly correlated with the basal‐like or classical subtype, (ii) be expressed specifically in the corresponding molecular subtype, (iii) be expressed only by tumor cells and not by stromal cells, and (iv) have available antibodies corresponding to the encoded protein that produce a staining compatible with routine use (intensity, ease of interpretation, robustness).

The first step was to perform an *in silico* marker selection. To achieve this selection, we performed a literature review to identify surrogate markers of classical and basal subtypes. References for this review were identified through searches of PubMed with the terms ‘pancreatic cancer’ and ‘molecular subtypes’, from 1995 until September 2023. Only papers published in English were reviewed. Articles were also identified through searches of the authors' own files. Ultimately, we identified nine markers for the classical subtype (GATA6, TFF1, CLDN18, HNF4A, HNF1A, REG4A, AGR2, KRT20 [CK20] and CDX2) and 15 for the basal subtype (MET, KRT5 [CK5], KRT6A [CK6A], KRT14 [CK14], S100A2, TP63, SNAI2, KRT17 [CK17], EGFR, SLC29A1 [ENT1], SLC16A3 [MCT4], SLC2A1 [GLUT1], MUC16, CYP24A1 and HMGA2) [3, 20, 21, 22, 23, 24, 25, 26, 27, 28] (supplementary material, Table S2). SLC29A1 was excluded because of the absence of commercially available antibody, leading to a selection of 23 markers. In addition, we analyzed transcriptomic data from a retrospectively well‐characterized multicenter cohort of 309 patients (Cohort 1) to explore their positive and negative correlation with both subtypes according to Puleo and Purist signatures [12, 25] (Figure 1B). Some markers had a low correlation with both subtypes, including EGFR, CYP24A1, and HNF1A.

The second step was to ensure the tumor‐specific expression of the 23 markers to support their relevance for subtype distinction. Their corresponding antibodies were assessed on a TMA of eight and 13 cases selected from Cohort 1 with homogeneous and strong basal‐like and classical signatures, respectively. The goal of this step was to select a robust antibody to detect only one subtype, but never the opposite subtype. Therefore, the selection of each antibody was based on a scientific approach promoting specificity over sensitivity by meeting three conditions: (i) its major expression in one subtype (i.e. at least 50% giving moderate/strong expression), (ii) its lack of expression in the opposite subtype (i.e. less than one strong expression or less than two moderate expressions), and (iii) its restricted expression in tumor cells (i.e. no expression in stromal cells). For more convenience, we used one cocktail that combined three markers (KRT5, KRT14, and p63), called PanBasal (PanBS). Representative examples of stainings are presented in supplementary material, Figure S2. The REG4 antibody that we tested strongly stained tumor‐associated neutrophils, and the CYP24A1 antibody gave weak or no staining, leading to the exclusion of these markers. The IHC expression of each candidate marker according to basal and classical signatures is shown in Figure 1C (except from REG4 and CYP24A1). We removed two classical (CDX2, KRT20) and two basal‐like markers (HMGA2, EGFR) because of their weak expression in the corresponding subtype in TMA. We also excluded five markers that showed expression in both tumor subtypes (AGR2, SNAI2, KRT6A, MET, SLC16A3). Finally, five classical markers (GATA6, CLDN18, TFF1, HNF1A, HNF4A) and four basal‐like markers (S100A2, KRT17, MUC16, PanBS) were selected because they were highly expressed and specific to the corresponding subtype.

The first step of selection on transcriptomic data was made from bulk analyses mixing RNAs from tumor and stromal cells and was also potentially influenced by the tumor cellularity. To assess tumor epithelial‐specific gene expression, we compared expression of our markers in ductal cell type 2 (PDAC cells) and in other cells (stroma) from public single‐cell data [18]. Except for SNAI2, all markers were significantly expressed in tumor cells (supplementary material, Figure S3A). To address the issue of stroma contamination, we used transcriptomic data from 30 murine xenografts of human PDAC (PDX) in which stromal (murine) and tumor (human) expression level could be distinguished [13]. We assessed the expression of selected genes in the tumor compartment according to the basal‐like and classical molecular subtypes (Chan‐Seng‐Yue signatures, supplementary material, Figure S4). All nine ‘purified’ markers indeed positively and negatively correlated with the corresponding and opposite subtype in PDX (*p* < 0.05).

The final step was to validate the selected markers in a large set of 50 human resected PDACs (Cohort 2). As tumor subtypes are based on RNA levels, we evaluated the concordance between the IHC expression and the corresponding mRNA level obtained by RNA‐seq. The reproducibility of our IHC scoring (H‐score) was validated through a double‐blind scoring of each marker in 15 tumors (supplementary material, Figure S5). IHC and RNA‐seq were performed on the same tumor area on the same tissue block (supplementary material, Figure S6). There was a strong and significant correlation (*p* < 0.001) between IHC expression level and mRNA transcript level for TFF1 (*r* = 0.87), CLDN18 (*r* = 0.82), S100A2 (*r* = 0.78), KRT17 (*r* = 0.74), PanBS (*r* = 0.62), MUC16 (*r* = 0.59), and GATA6 (*r* = 0.56). The association was nonsignificant for HNF4A (*r* = 0.19, *p* = 0.2) and HNF1A (*r* = 0.18, *p* = 0.22) and led us to remove these markers. Single‐cell data validated the removal of these two markers as they did not correlate with classical and basal signatures according to Chan‐Seng‐Yue [3] (supplementary material, Figure S3B). Finally, the validated markers were GATA6, CLDN18, and TFF1 for the classical subtype and MUC16, S100A2, KRT17, and PanBS for the basal‐like subtype.

Overall, this multistep process allowed us to conduct a thorough and rational selection of markers to differentiate the classical and basal‐like subtypes of a tumor. Our final selection of markers showed a clear negative correlation between the two groups within this cohort (Figure 1D). However, while very specific, not a single marker has enough sensitivity to be used alone to define the two molecular subtypes (Figure 1B,C). This observation supports the need for a combination of markers rather than one specific marker to discriminate both subtypes. This also strengthens a nondichotomous classification and an intratumor heterogeneity, which our marker panel might help to explore.

### Four types of tumoral clusters are revealed using unsupervised tile clustering

**Figure 2:**
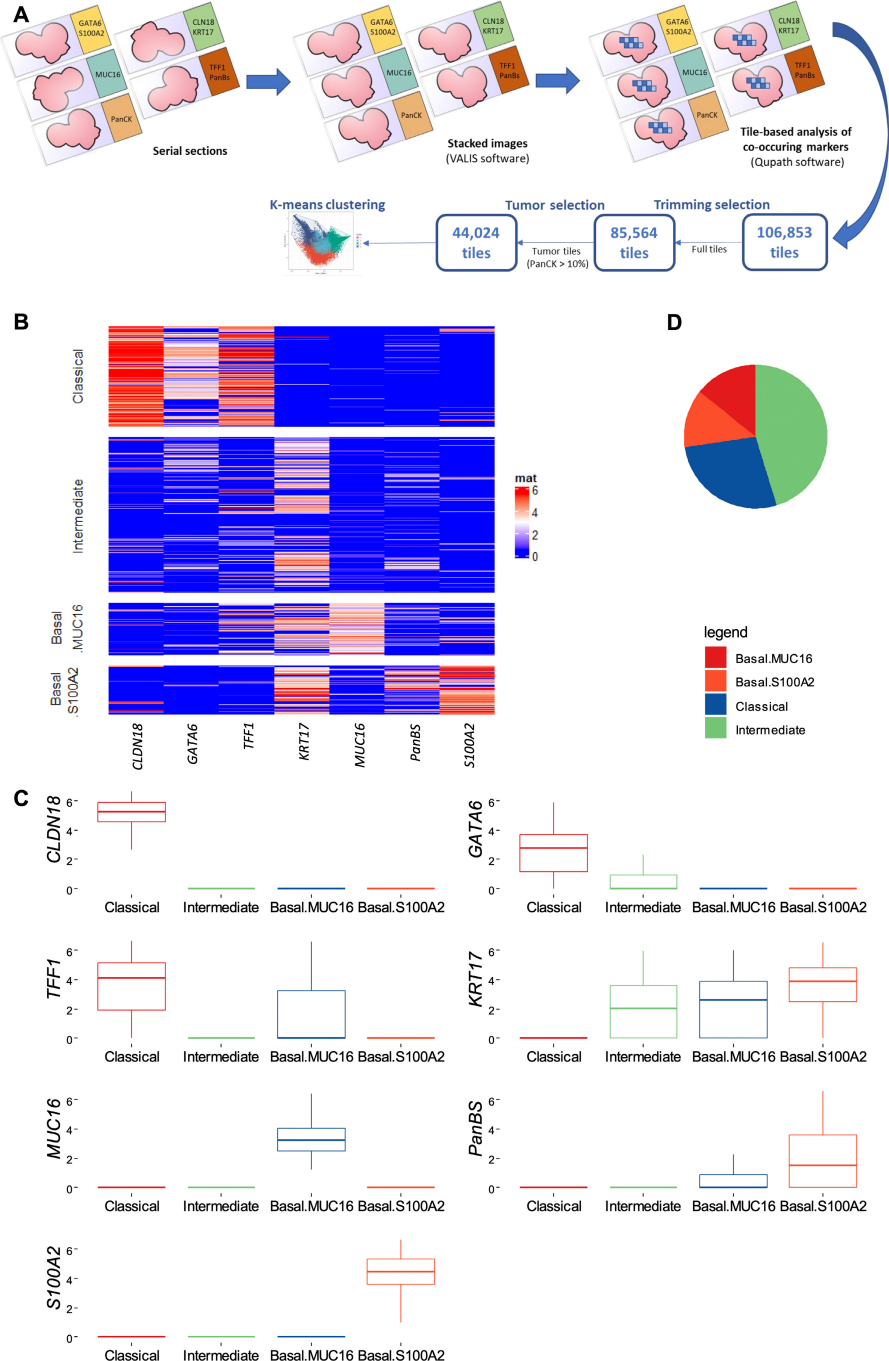
Tile clustering to explore pancreatic intratumor heterogeneity. (A) Stacked multiplex immunohistochemistry for intratumor subtype discovery using a tile‐based approach. PDAC: pancreatic ductal adenocarcinoma. Rotated profiles are shown to highlight lack of superposition between each slide when they are digitized, hence the need for registration (VALIS). (B) Heatmap representing marker IHC expression, as measured by proportion of positive pixels per tile (log2 transformed), according to clusters in Cohort 2. Rows are tumor tiles (*n* = 44,024). (C) Boxplots representing marker expression according to clusters in 95 resected PDACs (Cohort 2). (D) Pie chart representing proportions of clusters in whole slides from 95 resected PDACs (Cohort 2).

To assess the intratumor heterogeneity by the spatial co‐expression of the selected markers, we aligned digitized whole‐slide images corresponding to the H&E staining and the IHC (PANCK, GATA6/S100A2, CLDN18/KRT17, TFF1/PanBS, MUC16) (Figure 2A). We used this approach massively on a cohort of 95 patients with resected PDAC (Cohort 2). Tumor regions were manually annotated by an expert pathologist (JC) on H&E‐stained images. Each annotation was then copied on the serial IHC slides and was virtually cut into 200‐μm square tiles registered (i.e. aligned) on each slide. Positively stained pixels (PANCK, TFF1, CLDN18, GATA6, KRT17, MUC16, PanBS, and S100A2) were quantified to obtain a scoring proportion of each marker for each tile. The 106,853 tiles coming from all patients were filtered in a two‐step process in order to keep first the nontruncated (85,564) tiles and then the tumor (44,024) tiles containing more than 10% of PanCK staining.

To validate our marker panel, we evaluated the correlation of each marker with the classical and basal definition according to Moffitt [24] (supplementary material, Figure S7). Classical markers correlated positively with the classical signature and negatively with the basal signature (*p* < 0.05), and *vice versa* for basal markers, except for MUC16, which did not significantly negatively correlate with the classical signature (*p* = 0.30) but did strongly correlate with the basal signature (*p* < 0.0001). This observation encouraged us to continue further with our marker panel.

K‐means clustering of tiles using the proportion of positive pixels of the seven subtype markers revealed four types of tiles (supplementary material, Figure S8). Clusters according to marker expression are shown in Figure 2B,C. We found two basal clusters, including one with a predominant expression of MUC16, named Basal.MUC16, and one with a predominant expression of S100A2, named Basal.S100A2. PanBS was more expressed in the Basal.S100A2 cluster compared to the Basal.MUC16 cluster. A third cluster was typically classical with a mixed expression of CLDN18, GATA6, and TFF1 and the fourth cluster was negative for all markers except for KRT17 and to a lesser extent for GATA6 and was called intermediate. By pooling all tiles, basal, classical, and intermediate clusters represented 27.3%, 27.4%, and 45.3%, respectively (Figure 2D). Examples of representative areas of each cluster are depicted in supplementary material, Figure S9.

**Figure 3:**
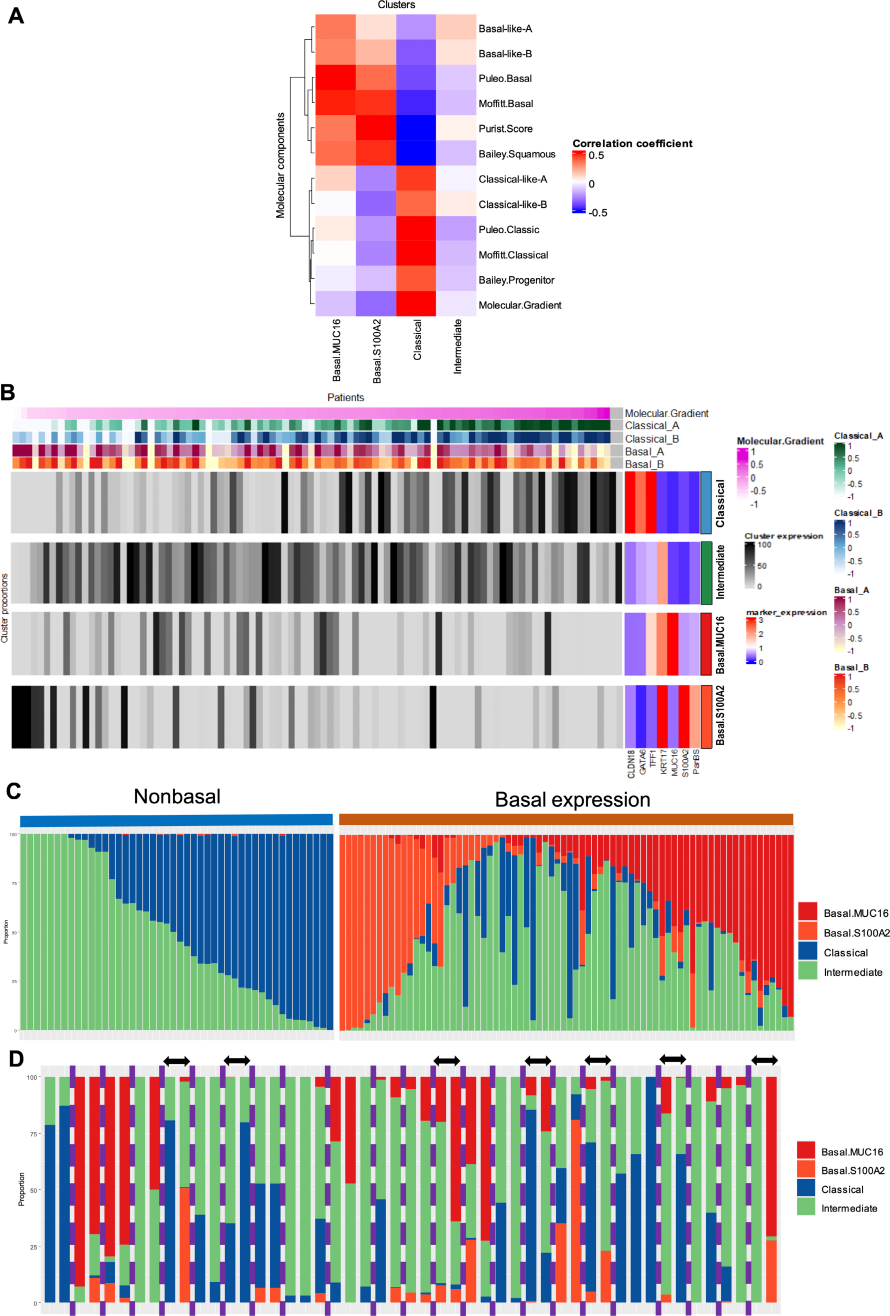
Representation of tile clusters and association with molecular subtypes. (A) Heatmap representing correlation of tile‐cluster proportions with transcriptomic‐based subtypes in Cohort 2. (B) Heatmap representing proportion of each cluster according to molecular gradient and Chan‐Seng‐Yue signatures in Cohort 2. Mean marker expression according to each cluster is represented on right side. (C) Bar plots representing tile‐cluster proportions in each tumor. Tumors are split between basal expression (>1% of tiles) and nonbasal expression (≤1% of tiles). (D) Bar plots representing per‐slide tile‐cluster proportions for each tumor with multiple slides (*n* = 23). Slides from different tumors are separated by a purple vertical dashed bar. Arrows represent shift in predominant cluster in the same tumor.

To validate these findings, we correlated each cluster expression with the previously defined molecular signatures (Figure 3A). Basal.S100A2 and Basal.MUC16 expression correlated with basal‐like signatures (Puleo, Moffit, Bailey, Purist Score) (*p* < 0.001). In contrast, the classical cluster correlated with classical signatures (*p* < 0.001) and the intermediate cluster did not correlate with either basal or classical signatures (*p* > 0.05). The expression of clusters in the 95 PDACs according to the molecular gradient [8] and Chan‐Seng‐Yue [3] signature is represented in Figure 3B. Classical tumors are also highly classical according to Chan‐Seng‐Yue, and their hybrid group (i.e. not classified as basal or classical) corresponds to intermediate tumors or to low expression of classical/basal in our cohort.

The proportion of each cluster by their major component is shown in Figure 3C. The intermediate cluster was almost always present (96%) and was predominant in 41% of PDACs. The mix of classical, basal, and intermediate clusters was present in 62.3% of cases, highlighting the important and constant intratumor heterogeneity of PDAC. To explore the spatial conservation of this heterogeneity, we analyzed spatially distinct areas from two different blocks from the same PDAC in 23 patients (Figure 3D). Interestingly, the predominant cluster remained the same for 16 PDACs (70%), highlighting the conserved heterogeneity in space.

To explore whether our marker panel was also relevant in metastatic patients, we compared RNA expression from 106 matched biopsies in primary tumor and liver metastases in 53 treatment‐naïve patients with metastatic PDAC (Cohort 4, supplementary material, Figure S10A). The classical genes *CLDN18* and *TFF1* were more expressed in the primary tumors compared to the liver metastases (*p* = 0.03 and *p* = 0.04, respectively), whereas other markers were equally expressed. The concordance at the protein level was verified by performing dual IHC (GATA6/S100A2) in one case with differential expression and two cases with equivalent RNA expression between the primary tumor and the liver metastasis (supplementary material, Figure S10B).

### The presence of a basal contingent sharply reduces survival, regardless of the remaining PDAC composition

**Figure 4:**
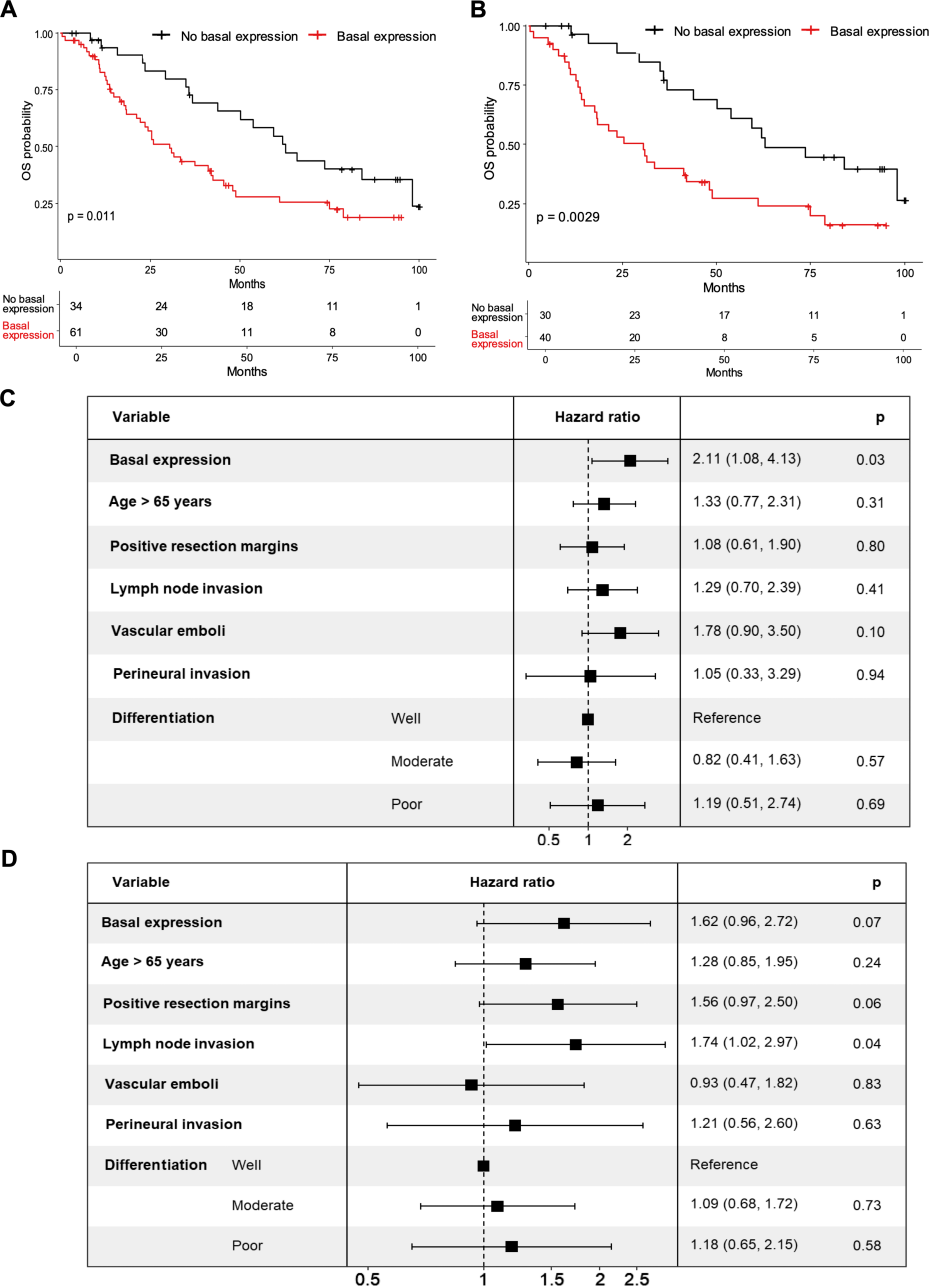
Survival of patients with PDAC according to cluster expression. (A) Overall survival curves according to detection of basal‐expressing tiles (>1% of tiles) in patients with PDAC (*n* = 95 patients, Cohort 2). OS: overall survival. (B) OS curves according to basal expression (>1% of tiles) in patients with classical‐expressing (>1% of tiles) PDAC (*n* = 70 patients, Cohort 2). OS: overall survival. (C) Multivariate analysis for detection of basal‐expressing tiles (>1% of tiles) and clinicopathological factors regarding OS (*n* = 95 patients, Cohort 2). (D) Multivariate analysis for basal expression (H‐score MUC16 or S100A2 > 0) and clinicopathological factors regarding OS (*n* = 148 patients, Cohort 3).

To assess the importance of assessing intratumor heterogeneity, we then evaluated whether the cluster expression among the 95 patients had a prognostic impact (Cohort 2). In multivariate analyses, neither the presence (>1%) nor the predominance (>50%) of the intermediate cluster impacted OS (*p* > 0.50) (supplementary material, Figure S11). Only the predominance of the classical cluster was associated with a trend of better OS (HR = 0.59, *p* = 0.10). In contrast, patients with PDAC containing even a minor basal (>1%) component (Basal.MUC16 and/or Basal.S100A2) had a strong decreased OS in univariate (median OS 30.5 months versus 62.8 months, *p* = 0.002) and multivariate analyses (HR = 2.36, *p* = 0.01) (Figure 4A–C). This prognostic association was also observed in an independent cohort of 148 patients with PDAC (Cohort 3) on TMA (H‐score > 0 regarding S100A2 or MUC16) in univariate (median OS 33.1 months versus 50.6 months, *p* = 0.04) and multivariate analyses (HR = 1.61, trend with *p* = 0.07) (Figure 4D). Of note, a TMA captures only one part of the tumor, and this could explain the trend rather than the significance by missing basal‐like cells in some cases. The basal expression as a continuous variable was not associated with OS in Cohort 2 (*p* = 0.40).

In addition, when restricting the analysis to patients with PDAC (Cohort 2) expressing the classical cluster (>1%), the observed difference in OS was even greater based on the co‐occurrence of a basal component (*p* < 0.001) (Figure 4B). Indeed, the group of patients with classical but no basal expression had a much longer OS in multivariate analysis (HR = 0.34, *p* = 0.003).

By exploring each basal cluster independently (Cohort 2), we found that the Basal.MUC16 cluster expression significantly and negatively impacted OS while a trend toward a decreased OS was observed for Basal.S100A2 cluster expression in multivariate analyses (HR = 1.93, *p* = 0.02; HR = 1.63, *p* = 0.08, respectively) (supplementary material, Figure S12A,B). Among patients with basal expression, OS was not impacted by the predominance of one of the basal clusters (*p* = 0.64) (supplementary material, Figure S12C). Similarly, OS among patients without basal expression was not impacted by the predominance of the classical or intermediate clusters (*p* = 0.91) (supplementary material, Figure S12D). In addition, none of the traditional clinicopathological parameters among T and N status, tumor differentiation, resection margins, and vascular and perineural invasion were significantly associated with a cluster expression (*p* > 0.05).

To ensure that our tile classification was not only recapitulating the histological differentiation, we assessed the basal‐like expression in IHC in well, moderately, and poorly differentiated tumors separately in the two surgical validation cohorts (supplementary material, Table S3). In both cohorts used for survival analysis (Cohorts 2 and 3), there were basal‐expressing tumors in the well‐differentiated group and no basal expression in some cases of the poorly differentiated group. In the whole‐slide cohort, basal expression was related to differentiation, but there was no complete redundancy. In the TMA‐based cohort, basal‐expression was not related to differentiation. Since TMAs only provide a view of a tiny portion of a tumor and we have shown that they are highly heterogeneous, the degree of differentiation estimated from the TMA may not accurately reflect the main differentiation pattern of the whole tumor. This reflects the shortcoming of employing TMAs in a heterogeneous neoplasm. Basal and classical markers were found expressed in well and poorly differentiated areas, respectively (supplementary material, Figure S13). In addition, our tile classification remained an independent prognostic factor from the differentiation in the multivariate analysis (Figure 4C,D). Overall, the presence of the basal phenotype was the most important prognostic factor, even in PDACs expressing the classical cluster.

### The evolutionary process of pancreatic carcinogenesis may start from a classical and progress toward a basal phenotype by going through an intermediate phenotype

**Figure 5:**
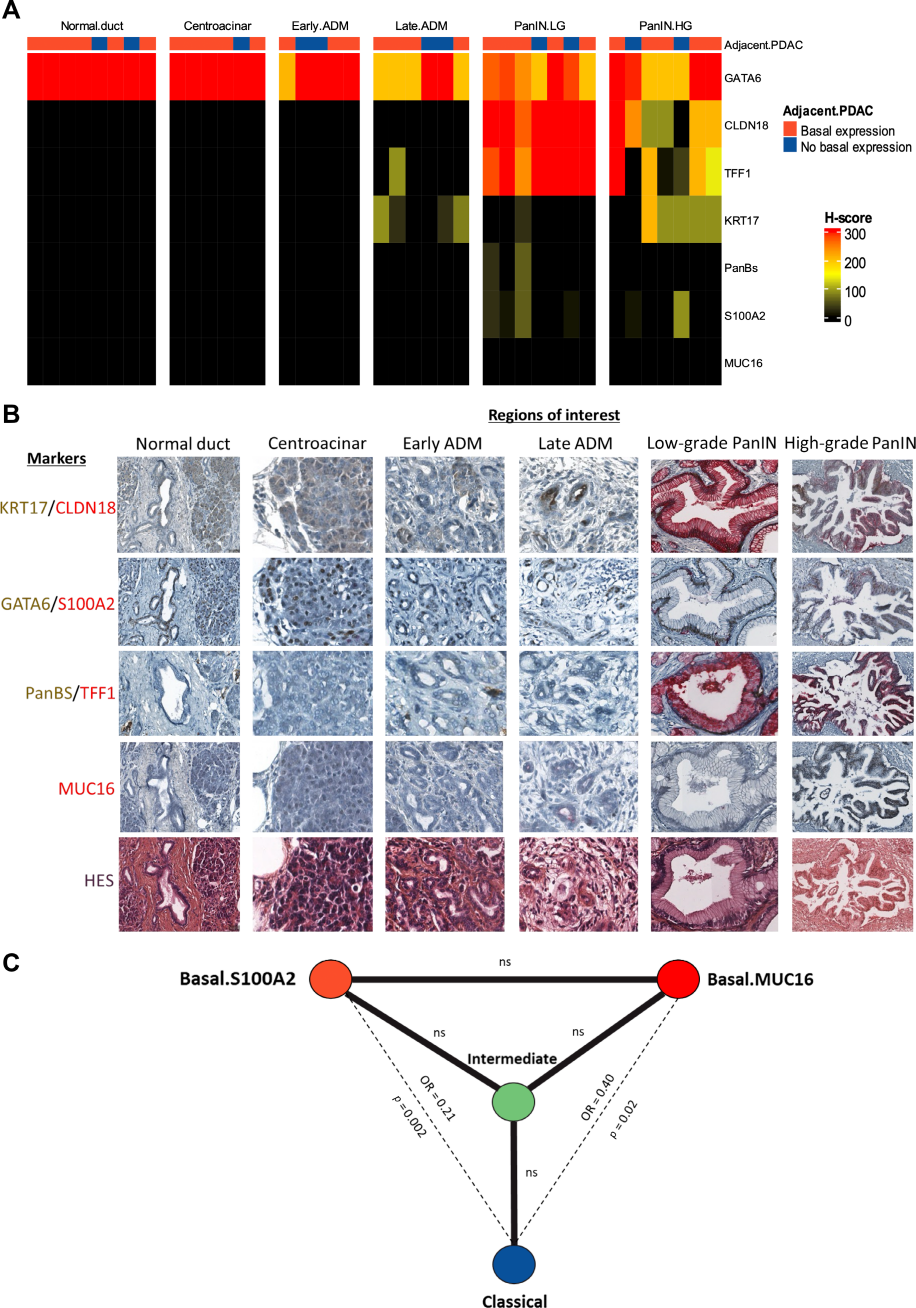
Marker expression in preneoplastic lesions and co‐occurrence between clusters among tumor tiles. (A) Heatmap representing marker expression in normal cells and preneoplastic lesions. ADM, acinar‐to‐ductal metaplasia; HG, high grade; LG, low grade; PanIN, pancreatic intraepithelial neoplasia. H‐score: 0 (black) to 300 (red). Each column represents one region of interest among normal duct, centroacinar, early and late ADM, LG and HG PanIN. The basal expression of an adjacent PDAC is represented in orange. (B) Marker expression according to regions of interest in representative areas. ADM: acinar‐to‐ductal metaplasia; HG: high grade; LG: low grade; PanIN: pancreatic intraepithelial neoplasia. KRT17, GATA6, and PanBS are in brown while CLDN18, S100A2, TFF1, and MUC16 are in red. Counterstain is H&E. (C) Cluster co‐occurrence among tumor tiles (Fisher's test).

Given that the previous steps showed the relevance of our marker panel, we wanted to explore which clusters are present at the first stages of pancreatic carcinogenesis. To do so, we studied the marker expression (H‐score) in preneoplastic lesions among 16 PDACs in Cohort 2 (four classical‐predominant, four basal‐predominant, and eight intermediate‐predominant), including five early acinar‐to‐ductal metaplasia (ADM), six late ADM, seven low‐grade pancreatic intraepithelial neoplasias (PanINs), and seven high‐grade PanINs. Fifteen normal areas were also analyzed (normal duct and centroacinar cells). Normal duct and centroacinar cells and early ADM strongly and exclusively expressed GATA6 (Figure 5A,B). Surprisingly, a moderate expression of KRT17 appeared in late ADMs and high‐grade PanINs along with a decrease in expression in classical markers in their early stage (early ADMs and low‐grade PanINs), independently of the basal expression in the adjacent PDAC. Other basal markers were weakly expressed or absent from normal and preneoplastic lesions. When expressed in PanINs, basal markers (KRT17, S100A2, and PanBS) were located at the basal cell pole, whereas classical markers (CLDN18, GATA6, TFF1) were at the apical pole (supplementary material, Figure S14). Taken together, these findings indicate the presence of a classical phenotype and KRT17, as an early basal marker, in preneoplastic lesions. Specific markers of basal clusters (i.e. MUC16 and S100A2) appear later during PDAC evolution, making them late basal markers.

At this stage, our hypothesis was that the Intermediate cluster with the predominant expression of KRT17 represented the missing link in a continuum between the classical and basal tumor cell phenotypes. Analyzing the co‐occurrence of phenotypes in each tumor, we observed that the intermediate cluster co‐occurred frequently with the other phenotypes, contrary to basal‐like and classical clusters (Figure 5C). This suggested that the intermediate cells may be a transitional state between the classical and basal clusters. Furthermore, six of the seven shifts (i.e. change in the predominant phenotype) observed in PDACs with more than one slide (*n* = 23) occurred between the intermediate and another cluster, and one between the classical and basal clusters (Figure 3D), reinforcing our starting hypothesis.

As co‐occurrence between both basal clusters was not less frequent than expected by chance (Figure 5C), we suspected two differential steps of evolution. To understand the biological substratum between the two basal clusters, we explored their differences. First, we observed that the six adenosquamous cases were all Basal.S100A2‐predominant, while none expressed more than 2% of Basal.MUC16. We then compared gene expression of PDACs with a major expression of Basal.MUC16 and less than 5% of Basal.S100A2 (*n* = 5) versus PDACs with a major expression of Basal.S100A2 and less than 5% of Basal.MUC16 (*n* = 6, including 4 out of six adenosquamous cases). First, genes involved in the classical program were decreased in the Basal.S100A2 cluster compared to the Basal.MUC16 cluster (supplementary material, Figure S15A), consistently with the previous correlations (Figure 3A) and suggesting a more distant state of differentiation. Second, the Basal.MUC16 cluster showed a significant increase (adjusted *p* < 0.05) in genes associated with bacterial infection (*PDZD3, SFTPA2, PIK3C2B*), cytokine signaling (*TRIM31, SERPINB2*), and apoptosis (*BCL2L15, VIL1*) (supplementary material, Figure S15B). The Basal.S100A2 cluster significantly upregulated genes associated with nervous system development (*IRX3, KRT6B, PKP1, ABC4, DSC3*), ECM modulation (*MMP13, COL7A1, PAK6*), and cell proliferation (*FAT2, S1PR5, GPC1, PTTG1*) (supplementary material, Figure S15B). GSEA validated the significant upregulation of proliferative pathways in the Basal.S100A2 cluster and inflammatory pathways in Basal.MUC16 cluster (supplementary material, Figure S15A). To validate these findings, we analyzed single‐cell data by comparing the top 5% of S100A2‐expressing cells and the top 5% of MUC16‐expressing cells (supplementary material, Figure S3D). Among the top differential genes, genes associated with cell proliferation (*FAT2, PCK1, SPDYC*), ECM modulation (*PODNL1, MMP10*), and nervous system development (*C1QL1, EN1*) were upregulated in S100A2 cells, while genes associated with bacterial infection (*IGJ*) and apoptosis (*CASP14, DIO3*) were upregulated in MUC16 cells. Proliferative pathways of S100A2 cells were highly enriched compared to MUC16 cells (*p* < 0.0001). To highlight the differences between the two subsets of basal cells, we selected the tumors with a high predominance (>80%) of one subset. We had a total number of 10 tumors including four MUC16 and six S100A2. While MUC16 tumors had a gland‐forming/cribriform morphology, S100A2 tumors were non‐gland‐forming/adenosquamous (supplementary material, Figure S16). To validate the link with the proliferation signatures obtained by RNAseq, we performed staining for Ki‐67 in these 10 tumors and calculated the proliferation index in tumor cells (Qupath‐based computer‐assisted Ki‐67 count) and found that proliferation was higher in S100A2 tumors (*p* = 0.04).

## Discussion

Herein, we developed a panel of antibodies that could easily be used by researchers and pathologists. The purpose of this panel was to classify patients according to the two main subtypes of PDAC, roughly basal‐like or classical. To achieve this, we selected markers through a stringent and multistep process. This highlighted the limit of RNA‐based selection as some genes such as *KRT6A* were linked to the epithelial component, regardless of the subtype, but appeared as strongly differential due to the tumor cellularity of basal‐like tumors being much higher than classical tumors. Current scientific knowledge of PDAC suggests that this cancer presents significant intertumor and intratumor heterogeneity. Our panel of seven markers (GATA6, CLDN18, TFF1, MUC16, S100A2, KRT17, PanBS) revealed the limits of the binary basal‐like/classical classification by highlighting the major intermediate cluster co‐expressing classical and basal‐like markers, which does not fit into this classification. In our study, the proportions of predominant basal‐like, classical, and intermediate tumors were 28%, 31%, and 41%, respectively. It differs from previously published studies where the rates were around 20%, 60%, and 20%, respectively [3, 12, 21, 22, 24]. In those studies, tumor subtypes were mostly assessed through bulk RNA, whereas we used a whole‐slide approach, which may explain the differences in proportions. Our previous study using a multistep deep learning model was also based on whole slides to predict the tumor subtypes and found approximately the same proportions as our current work did [2]. In addition, we demonstrated a high degree of intratumor heterogeneity, with a frequent co‐occurrence of classical, basal, and intermediate clusters, both between tumors and within the same tumor. The study by Chan‐Seng‐Yue *et al* was the first to suggest that PDACs may be a mix of basal and classical cells, with an unclassified subgroup of tumors referred to as hybrid [3]. More studies provided more evidence of tumor plasticity and demonstrated that this subgroup of cells could be an intermediate phenotype in transition between the classical and basal program [10, 29, 30]. However, the exact nature and definition of this intermediate phenotype are currently unknown. Our study reinforces the idea that an intermediary phenotype is present at the protein level and furnishes important information about the sequence of the transformation. KRT17 seems to be the first marker leading to the basal program after the loss of the classical program, at the crossroads of a further evolution toward S100A2 or MUC16 expression. However, the exact mechanism of this transition is unknown. Raghavan *et al* and Michiels *et al* suggested that the tumor microenvironment and, more specifically, cancer‐associated fibroblasts could modulate the transition of epithelial cells [30, 31].

This tremendous intratumor heterogeneity and likely subsequent plasticity could explain the treatment resistance and the very poor prognosis of PDAC. The prognosis of these patients comes from the metastases that will later develop and be predominantly basal. Our results suggest that a minor basal component is sufficient to increase the metastatic risk and ultimately impact the prognosis.

Our study shows the difficulty of the interpretation of PDAC heterogeneity using bulk transcriptomic approaches, thereby limiting their routine application. Several studies have identified biomarkers for routine tumor subtyping, but they were limited to just a few markers [20, 32, 33]. In accordance with previous studies [10, 31], our work supports the need for a panel of markers rather than single markers to address PDAC heterogeneity. Williams *et al* recently proposed a multiplex immunofluorescence panel including six of our markers [10] (CLDN18.2, TFF1, GATA6, KRT17, KRT5, and S100A2). Given the strong prognostic value of the basal expression and the distinct biology of the Basal.MUC16 cluster, adding MUC16 would be optimal to entirely capture the basal expression. MUC16‐expressing tumor cells are different from S100A2‐expressing tumor cells (supplementary material, Figure S9E), and if MUC16 were not part of our panel, about a third of basal‐expressing tumors would be missing (Figure 3C). Our group is leading the PRODIGE‐104 NeoPREDICT phase II trial, whose aim is to personalize neoadjuvant chemotherapy according to transcriptomic signatures in borderline PDAC. We plan to add the staining of at least one classical marker (e.g. CLDN18) and two specific basal markers (i.e. S100A2 and MUC16) to the ancillary program of this trial to easily refine patient prognosis and support their implementation in clinical practice. In the same way, a phase II trial (NeoPANCOne, NCT04472910) is currently evaluating the value of GATA6 (FISH and IHC) in chemotherapy resistance in resectable PDACs [34]. Facilitating the assessment of tumor heterogeneity could also be useful in the case of sequential tumor assessment under experimental treatment modulating PDAC phenotype (e.g. epigenetic strategies) [35].

Importantly, our study brings new insight into the presence and the possible co‐occurrence of two different basal phenotypes. Although the prognostic difference between both basal phenotypes was not apparent in our study, the information that a minor expression of one of the two was enough to drastically reduce OS is important and aligned with our previous work [2]. This highlights the importance of a complete tumor analysis, as some classical PDACs may harbor aggressive basal contingents.

Overall, our thorough selection of basal and classical markers classified 54.7% of tumoral tiles as basal or classical, but the remaining tiles had a nonclassical and a nonbasal phenotype. The intermediate phenotype was characterized by the predominant expression of KRT17, nonspecific to the basal subtype. This confirmed its intermediate nature, as reported in the single‐cell studies [10, 36], in which KRT17 was activated in cells that co‐expressed basal and classical markers. KRT17 could be expressed alone or with the late basal markers S100A2 and MUC16 that were exclusive of classical markers. Consistent with Williams *et al*, the intermediate state was almost always present in PDACs, showing its potential importance in the transition between classical and basal‐like subtypes. By analyzing preneoplastic lesions, we showed evidence of the evolution process of pancreatic carcinogenesis going from a classical toward a basal phenotype through an intermediate phenotype. This is consistent with the observation that the final stage of PDAC development (i.e. metastasis) is dominated by basal cells [10]. Sequential samples would help support this hypothesis, and the exact mechanisms of this transition (e.g. epigenetic remodeling) remains to be defined.

## Author contributions statement

JC and RN conceived the study. MH and FD curated the data. MH and RN analyzed the data. All authors were involved in writing the paper and had final approval of the submitted and published versions.

## Supporting information


**Figure S1.** Techniques and aims regarding the four cohorts of patients with pancreatic ductal adenocarcinoma
**Figure S2**. Example of labeling using the different antibodies tested in a typical classical PDAC (blue) and a typical basal PDAC (red)
**Figure S3**. Single‐cell data from Peng *et al*
**Figure S4**. Correlation of gene expressions with Chan‐Seng‐Yue signatures in tumor compartment of 30 patient‐derived xenografts
**Figure S5**. Double‐blind IHC scoring of selected markers in 15 tumors from Cohort 2
**Figure S6**. Correlation between marker expression levels in IHC (H‐score) and RNAseq in 50 pancreatic ductal adenocarcinomas from Cohort 2
**Figure S7**. Correlation of each marker with classical and basal definition according to Moffitt in 95 pancreatic ductal adenocarcinomas (Cohort 2)
**Figure S8**. K‐means clustering of 44,024 tiles from 95 pancreatic ductal adenocarcinomas (Cohort 2)
**Figure S9**. Examples of representative areas of each cluster
**Figure S10**. Expression of selected markers according to site of biopsy in matched samples from patients with treatment‐naïve metastatic PDAC (Cohort 4)
**Figure S11**. Survival according to cluster. Multivariate analysis for (A and B) classical expression and clinicopathological factors regarding overall survival (*n* = 95 patients) and (C and D) intermediate expression and clinicopathological factors regarding overall survival (*n* = 95 patients)
**Figure S12**. Survival according to cluster. Multivariate analysis for (A and B) basal expression and clinicopathological factors regarding overall survival (*n* = 95 patients). Overall survival curves according to predominant cluster in (C) basal‐expressing PDAC (*n* = 61 patients) and (D) basal‐free PDAC
**Figure S13**. IHC expression of MUC16, CLDN18, S100A2, and TFF1 in poorly and well‐differentiated areas
**Figure S14**. Expression of IHC panel in pancreatic intraepithelial neoplasia
**Figure S15**. Differential analysis between basal clusters
**Figure S16**. Morphology and proliferation comparison between basal clusters
**Table S1**. Characteristics of the 24 tested antibodies.
**Table S2**. References chosen from literature review for *in silico* marker selection of basal and classical subtypes
**Table S3**. Immunohistochemistry: basal expression according to tumor differentiation in Cohorts 2 and 3

## Data Availability

The data generated in this study are available upon request from the corresponding author. The data are not publicly available due to privacy or ethical restrictions.
