# Supplementary material for: Redefining phenotypic intratumor heterogeneity of pancreatic ductal adenocarcinoma: a bottom‐up approach
Source: J Pathol. 2025 Feb 11;265(4):448–61. doi: 10.1002/path.6398 (PMC11880971; doi:10.1002/path.6398)
Supplement: Supplementary file 1 — Figure S1. Techniques and aims regarding the four cohorts of patients with pancreatic ductal adenocarcinoma Figure S2. Example of labeling using the different antibodies tested in a typical classical PDAC (blue) and a typical basal PDAC (red) Figure S3. Single‐cell data from Peng et al Figure S4. Correlation of gene expressions with Chan‐Seng‐Yue signatures in tumor compartment of 30 patient‐derived xenografts Figure S5. Double‐blind IHC scoring of selected markers in 15 tumors from Cohort 2 Figure S6. Correlation between marker expression levels in IHC (H‐score) and RNAseq in 50 pancreatic ductal adenocarcinomas from Cohort 2 Figure S7. Correlation of each marker with classical and basal definition according to Moffitt in 95 pancreatic ductal adenocarcinomas (Cohort 2) Figure S8. K‐means clustering of 44,024 tiles from 95 pancreatic ductal adenocarcinomas (Cohort 2) Figure S9. Examples of representative areas of each cluster Figure S10. Expression of selected markers according to site of biopsy in matched samples from patients with treatment‐naïve metastatic PDAC (Cohort 4) Figure S11. Survival according to cluster. Multivariate analysis for (A and B) classical expression and clinicopathological factors regarding overall survival (n = 95 patients) and (C and D) intermediate expression and clinicopathological factors regarding overall survival (n = 95 patients) Figure S12. Survival according to cluster. Multivariate analysis for (A and B) basal expression and clinicopathological factors regarding overall survival (n = 95 patients). Overall survival curves according to predominant cluster in (C) basal‐expressing PDAC (n = 61 patients) and (D) basal‐free PDAC Figure S13. IHC expression of MUC16, CLDN18, S100A2, and TFF1 in poorly and well‐differentiated areas Figure S14. Expression of IHC panel in pancreatic intraepithelial neoplasia Figure S15. Differential analysis between basal clusters Figure S16. Morphology and proliferation comparison between basal clusters Tabl [file PATH-265-448-s001.docx]

**Redefining phenotypic intratumor heterogeneity of pancreatic ductal adenocarcinoma: a bottom-up approach**

M Hilmi *et al. J Pathol* <https://doi.org/10.1002/path.6398>

**Supplementary Figures S1–S16**

**Supplementary Tables S1–S3**

Reference numbers refer to the main text list

**
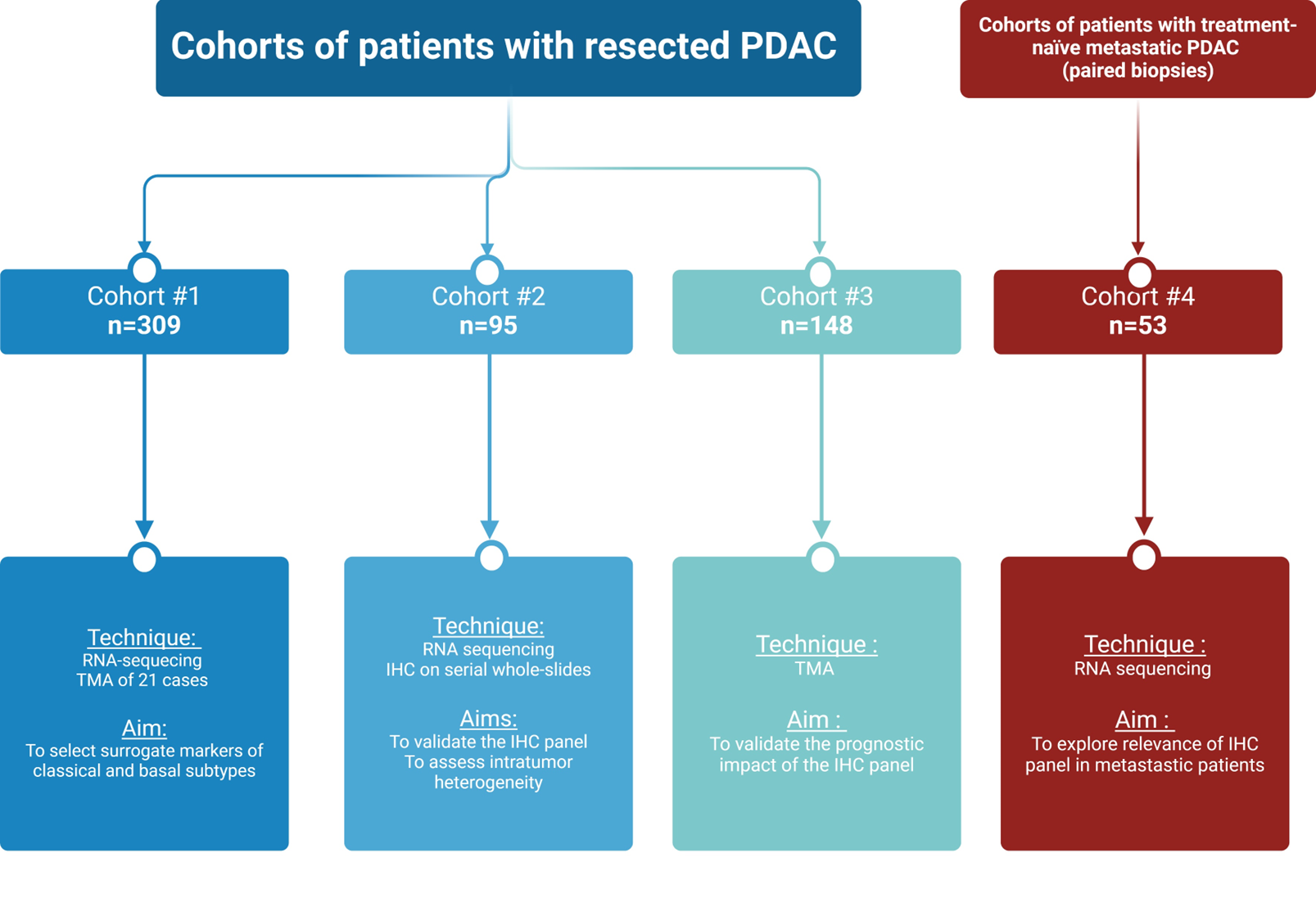
**

**Figure S1.** Techniques and aims regarding the four cohorts of patients with PDAC. Created with BioRender.com.


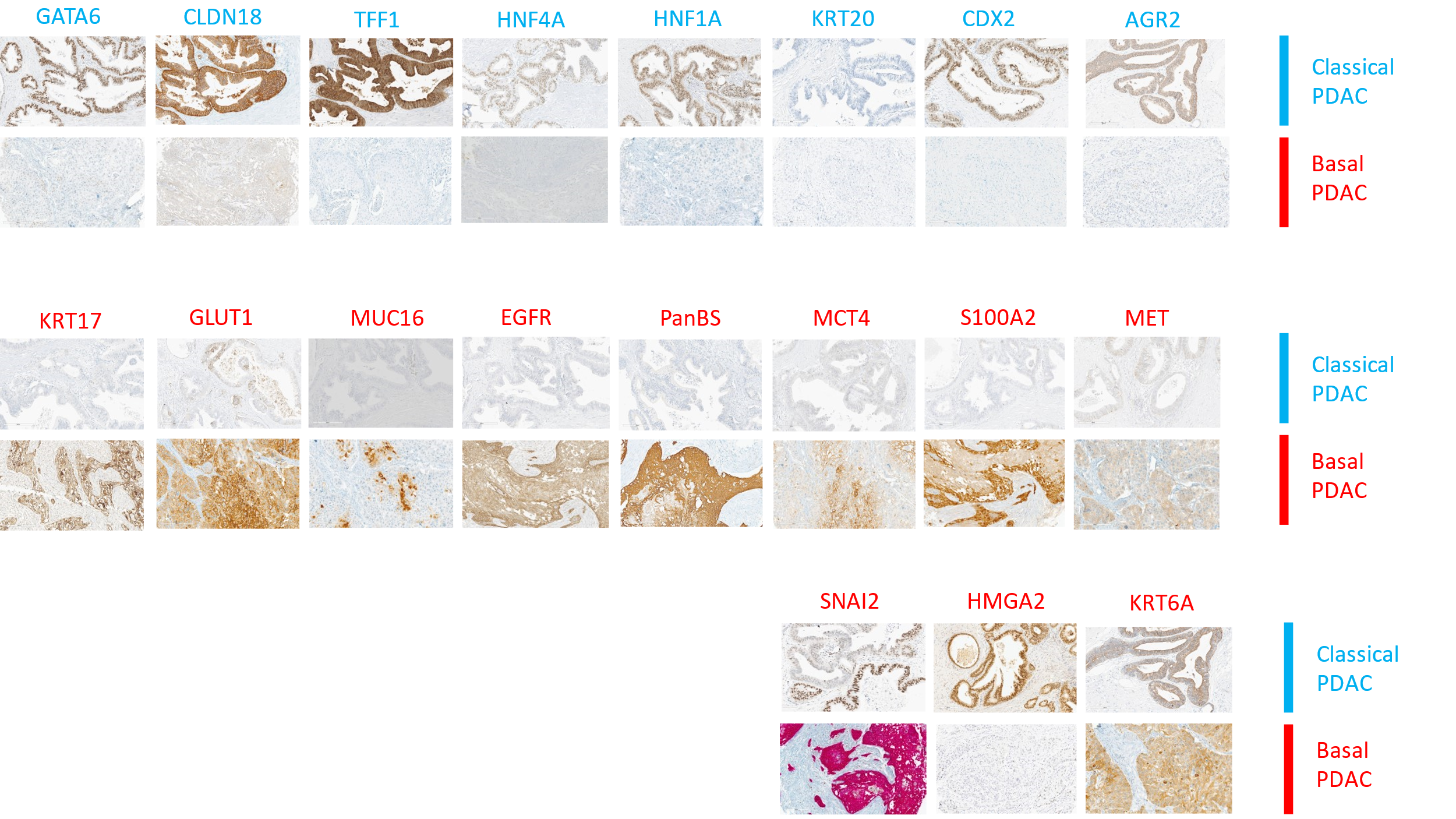


**Figure S2.** Example of labeling using the different antibodies tested in a typical classical PDAC (blue) and a typical basal PDAC (red). Classical markers are in blue, basal markers in red. All markers are in brown, except CK5 in purple, along with SNAI2 in brown.


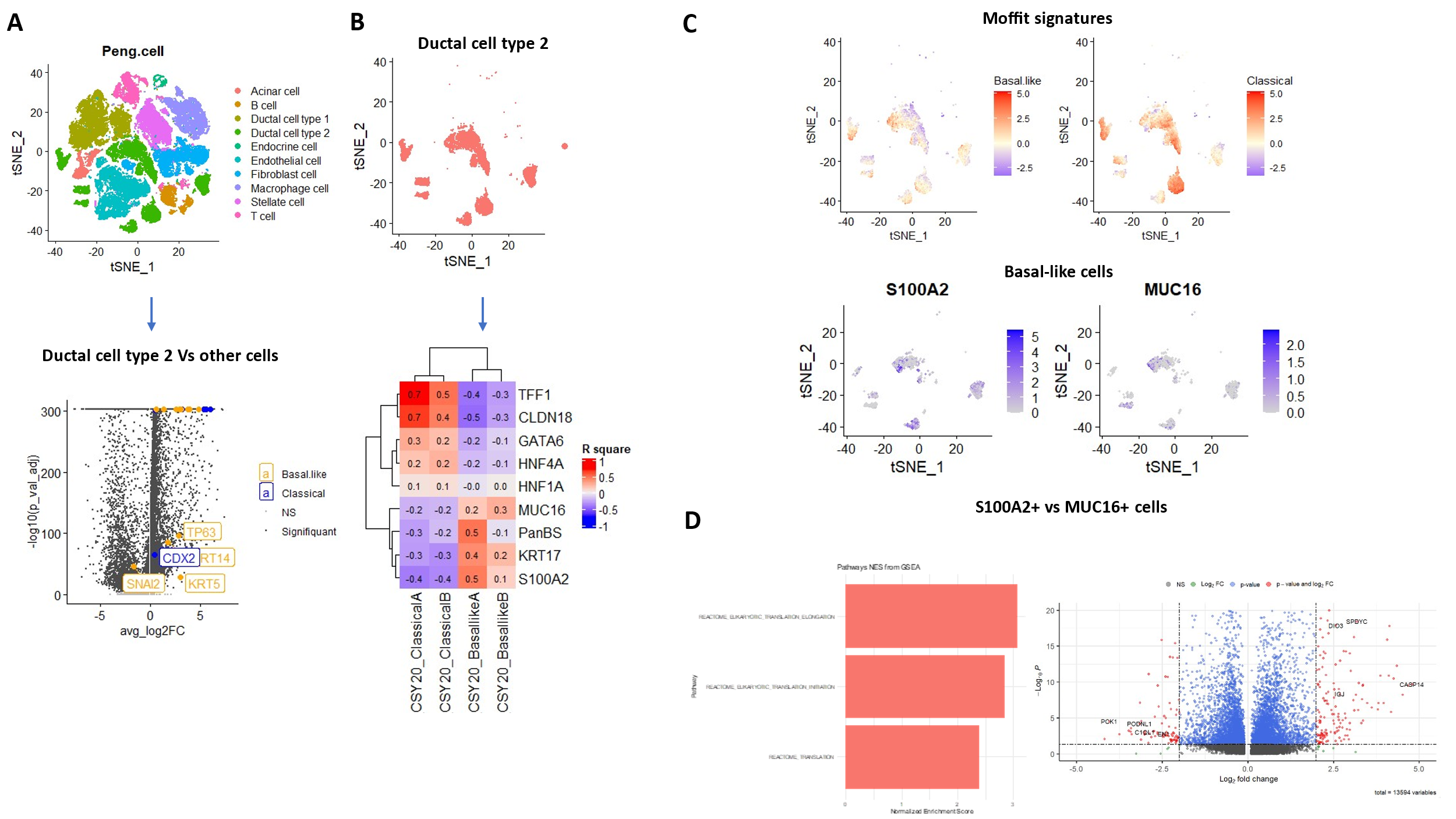


**Figure S3.** Single-cell data from Peng *et al* [18]. (A) Differential expression of nine selected genes. (B) Correlation of nine selected genes with Chan-Seng-Yue signature in ductal cell type 2. (C) *S100A2* and *MUC16* expression in basal-like cells. (D) GSEA analysis showing signaling pathways that are differentially regulated and statistically significant (adjusted *p* value < 0.05) between S100A2 and MUC16 cells and volcano plot showing differentially expressed genes between S100A2 and MUC16 cells (dashed lines indicate threshold of significant gene expression, defined as log2-transformed fold-change ≤ −2.0 and ≥ 2.0 with adjusted *p* value < 0.05).

**Figure S4.** Correlation of gene expressions with Chan-Seng-Yue signatures in tumor compartment of 30 patient-derived xenografts.

**Figure S5.** Double-blind IHC scoring of selected markers in 15 tumors from Cohort 2.


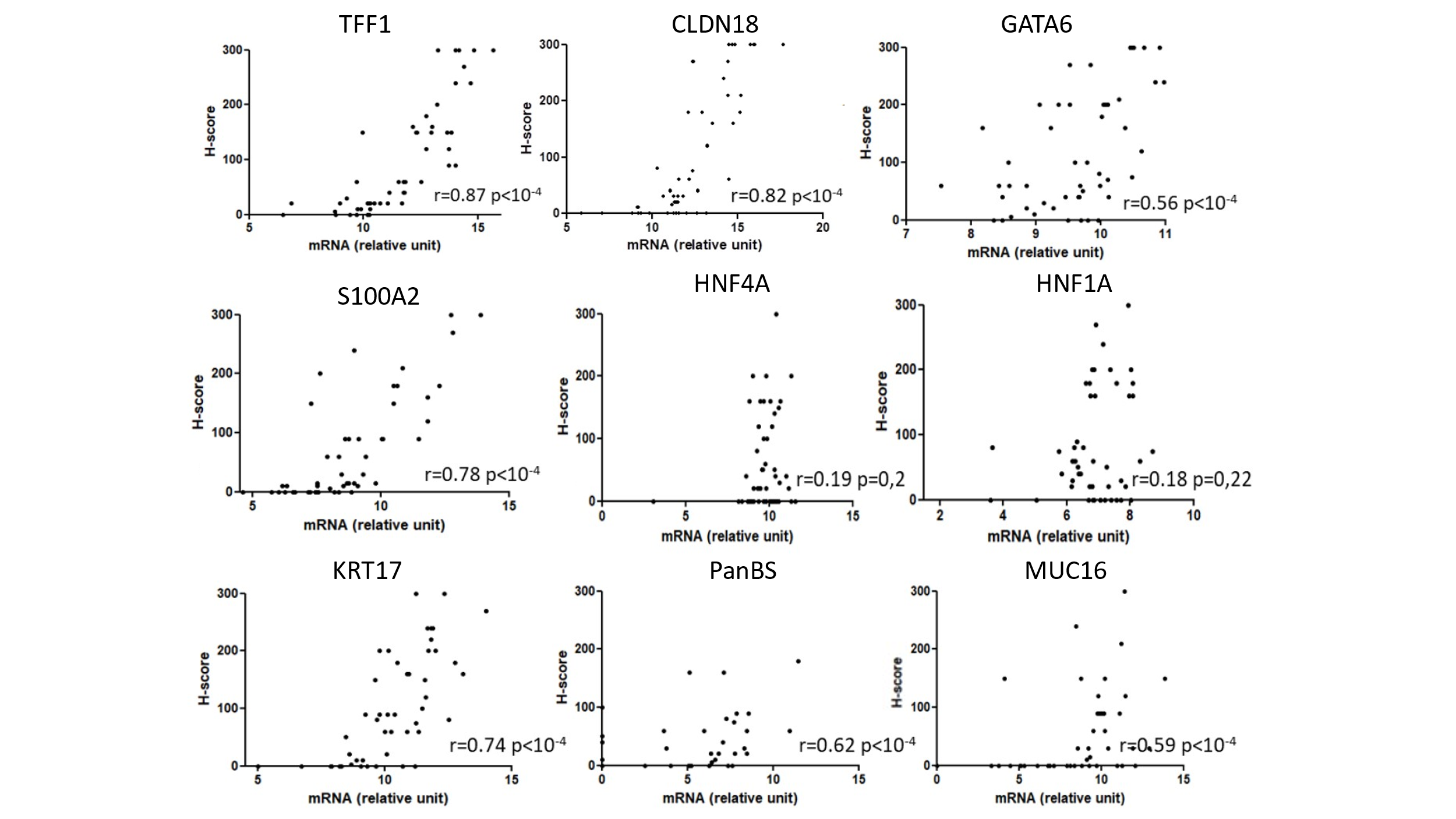


**Figure S6.** Correlation between marker expression levels in IHC (H-score) and RNA-seq in 50 PDACs from Cohort 2.


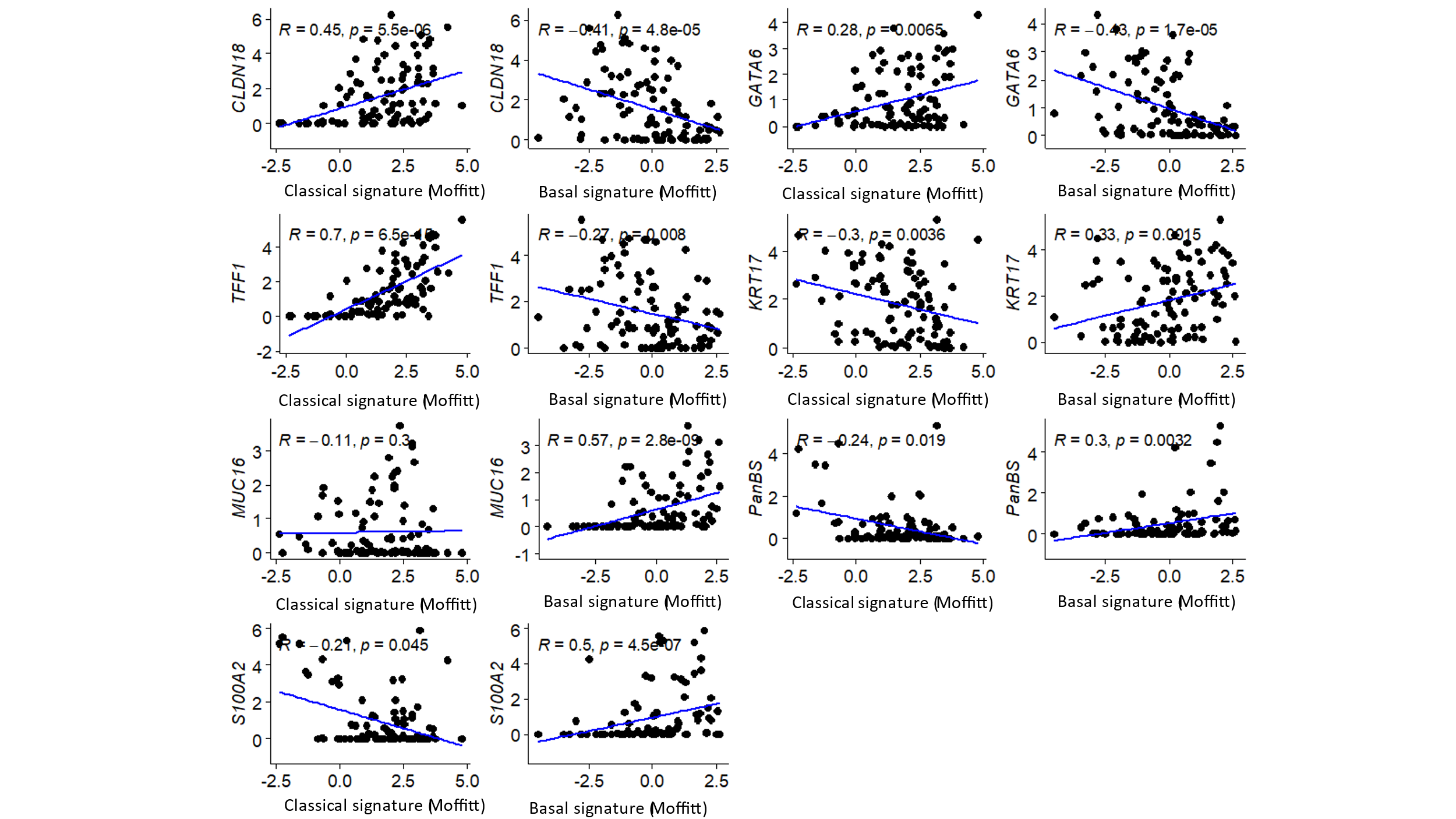


**Figure S7.** Correlation of each marker with classical and basal definition according to Moffitt *et al* [24] in 95 PDACs (Cohort 2).

**
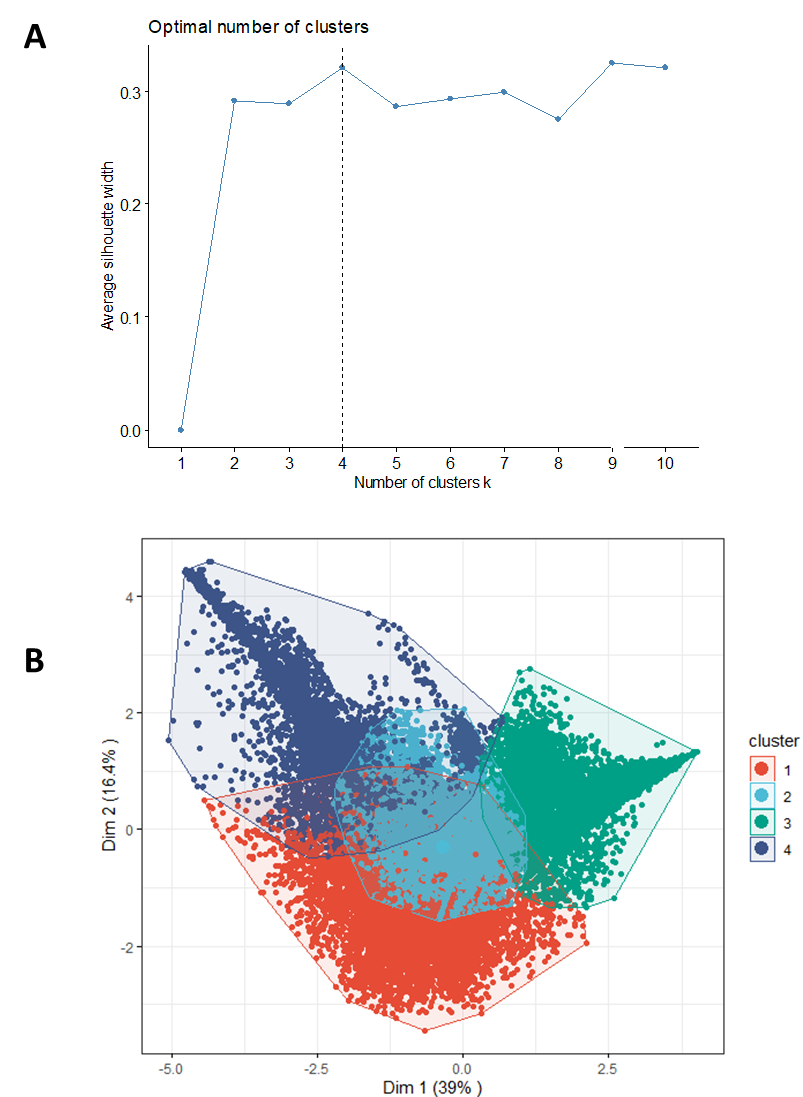
**

**Figure S8.** K-means clustering of 44,024 tiles from 95 PDACs (Cohort 2). (A) Number of clusters (k) according to average silhouette width. Black dashed line shows optimal number of clusters. (B) Visualization of the four clusters on the first two axes of principal component analysis.


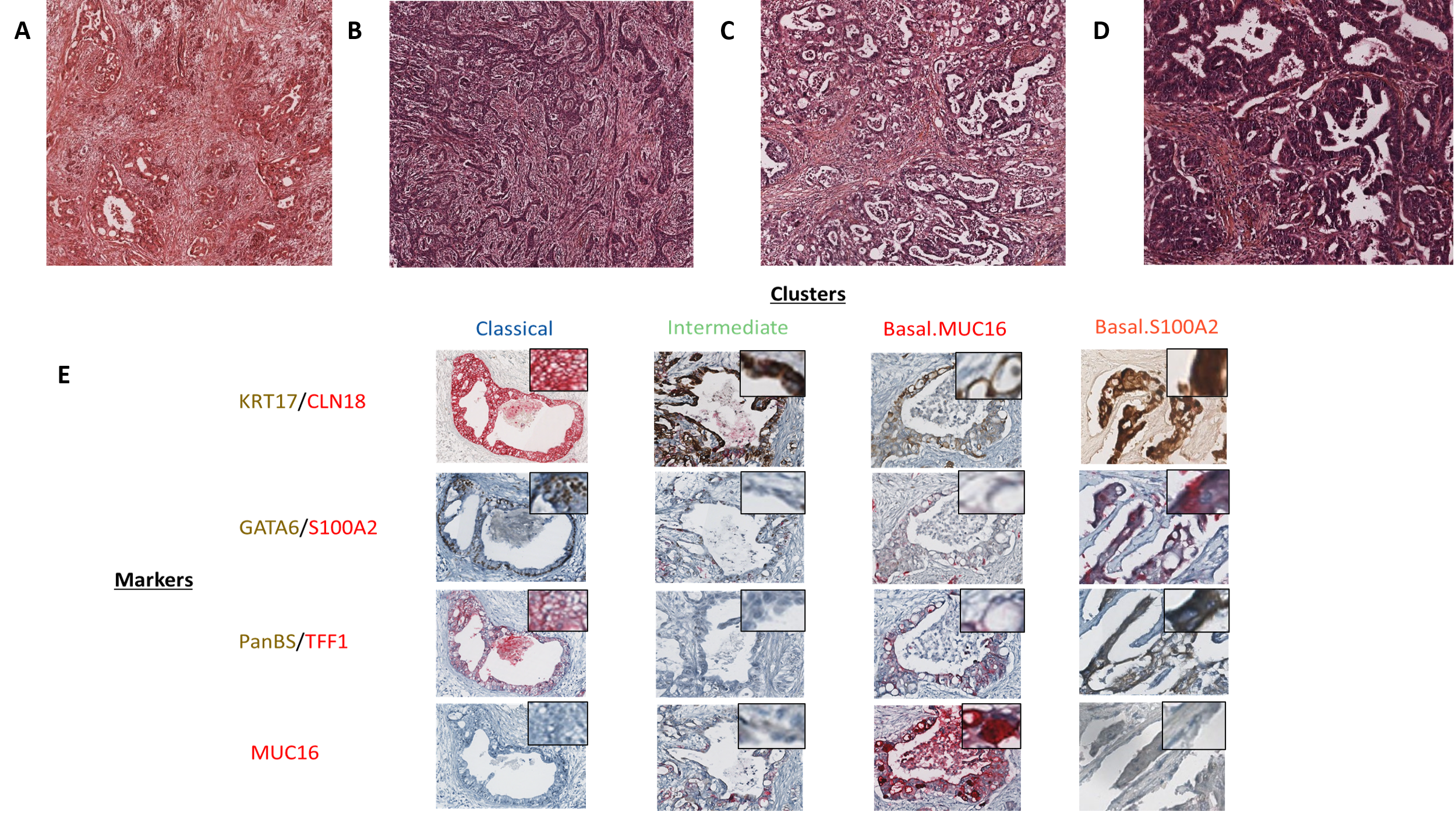


**Figure S9.** Examples of representative areas of each cluster. A = Basal.MUC16, B = Basal.S100A2, C = classical, D = intermediate, E = marker expression according to each cluster in representative areas. Zoom is incorporated at upper right of each image.


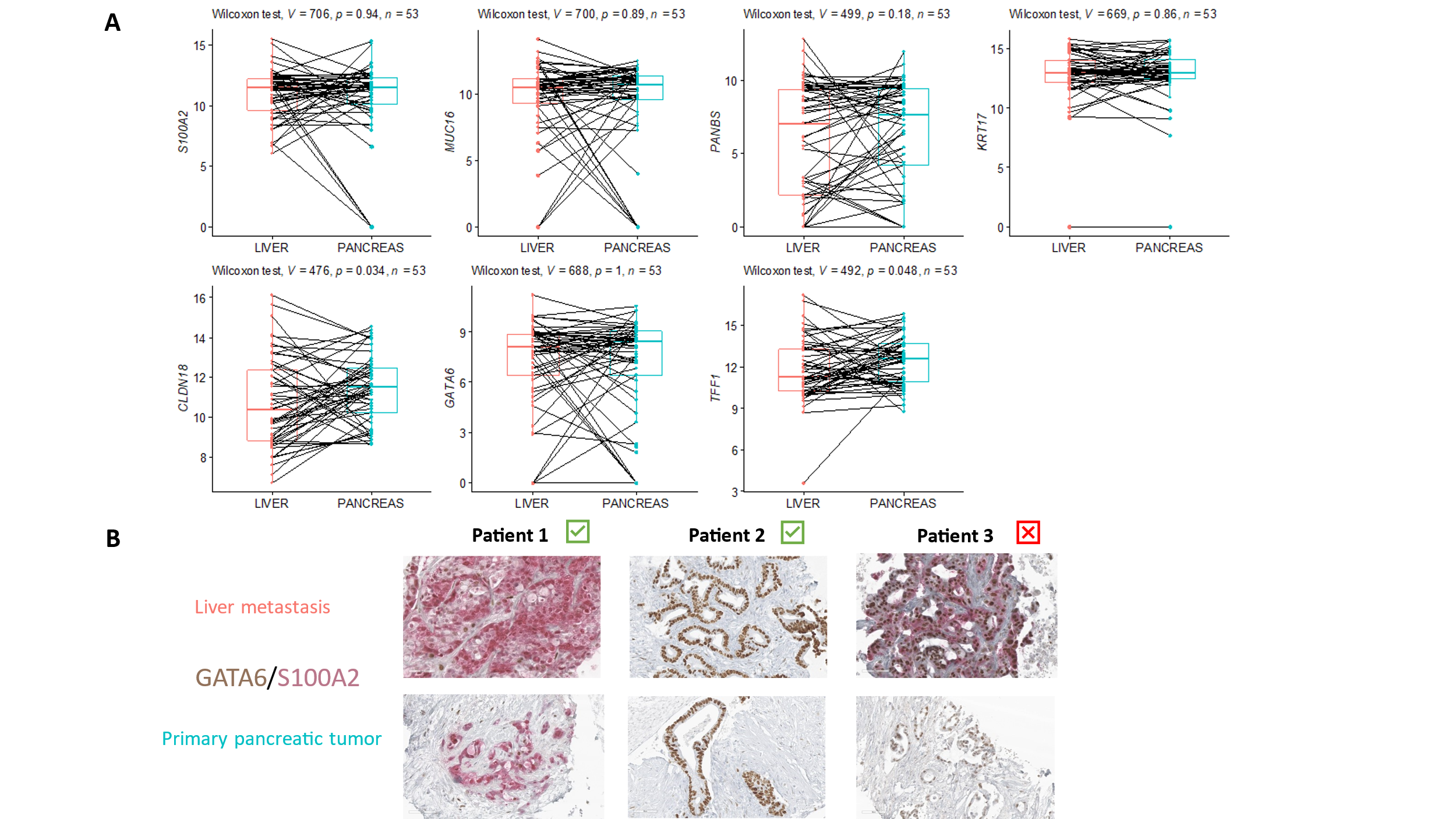


**Figure S10.** Expression of selected markers according to site of biopsy in matched samples from patients with treatment-naïve metastatic PDAC (Cohort 4). (A) Gene expressions in 106 matched samples from 53 patients. (B) GATA6 (brown) and S100A2 (red) IHC staining in matched biopsies. Patient 1: strong S100A2 expression in both biopsies. Patient 2: strong GATA6 expression in both biopsies. Patient 3: strong S100A2 expression in liver metastasis and low expression in primary tumor.


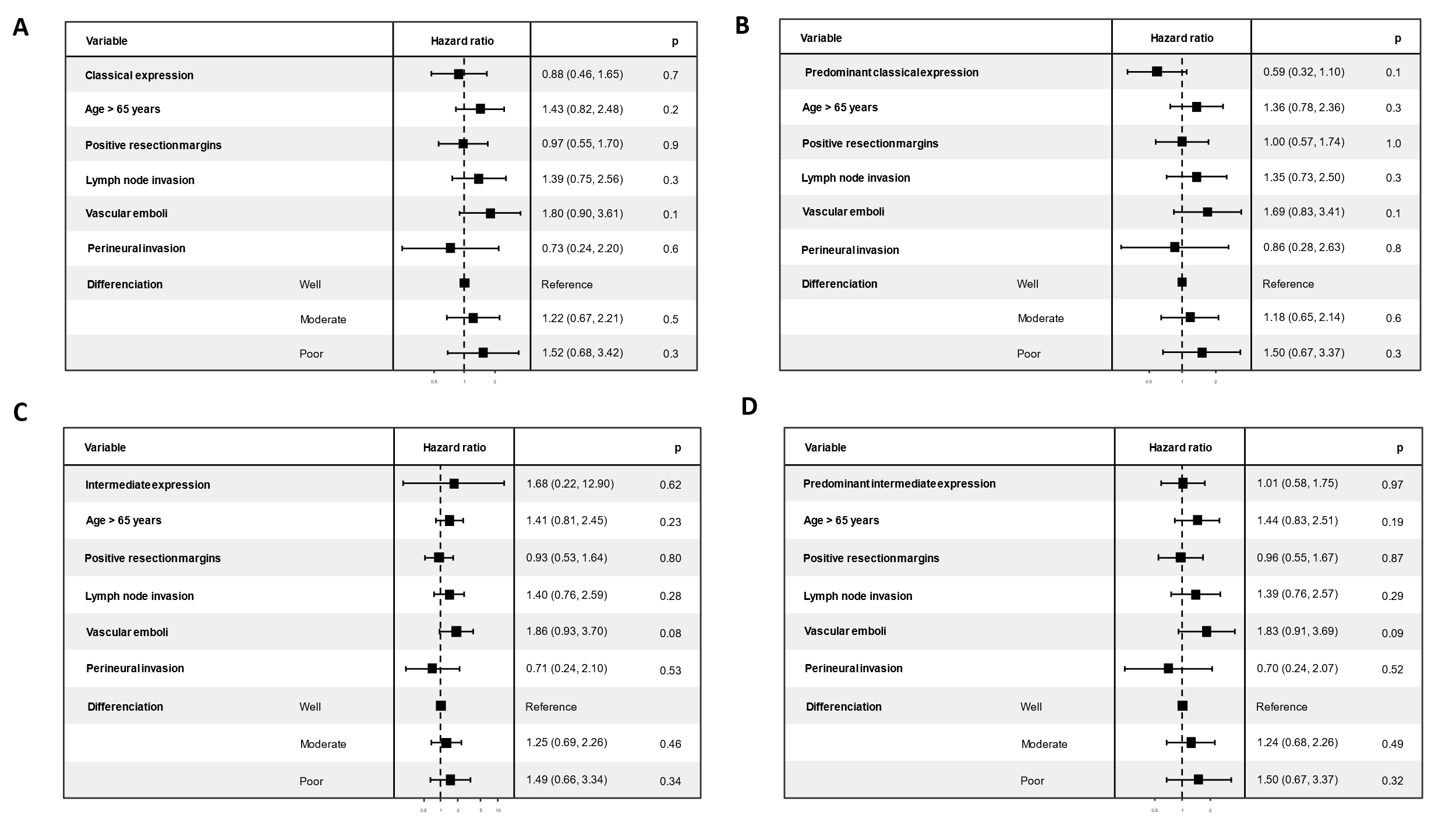


**Figure S11.** Survival according to cluster. Multivariate analysis for (A and B) the classical expression and clinicopathological factors regarding overall survival (*n* = 95 patients) and (C and D) multivariate analysis for intermediate expression and clinicopathological factors regarding overall survival (*n* = 95 patients).


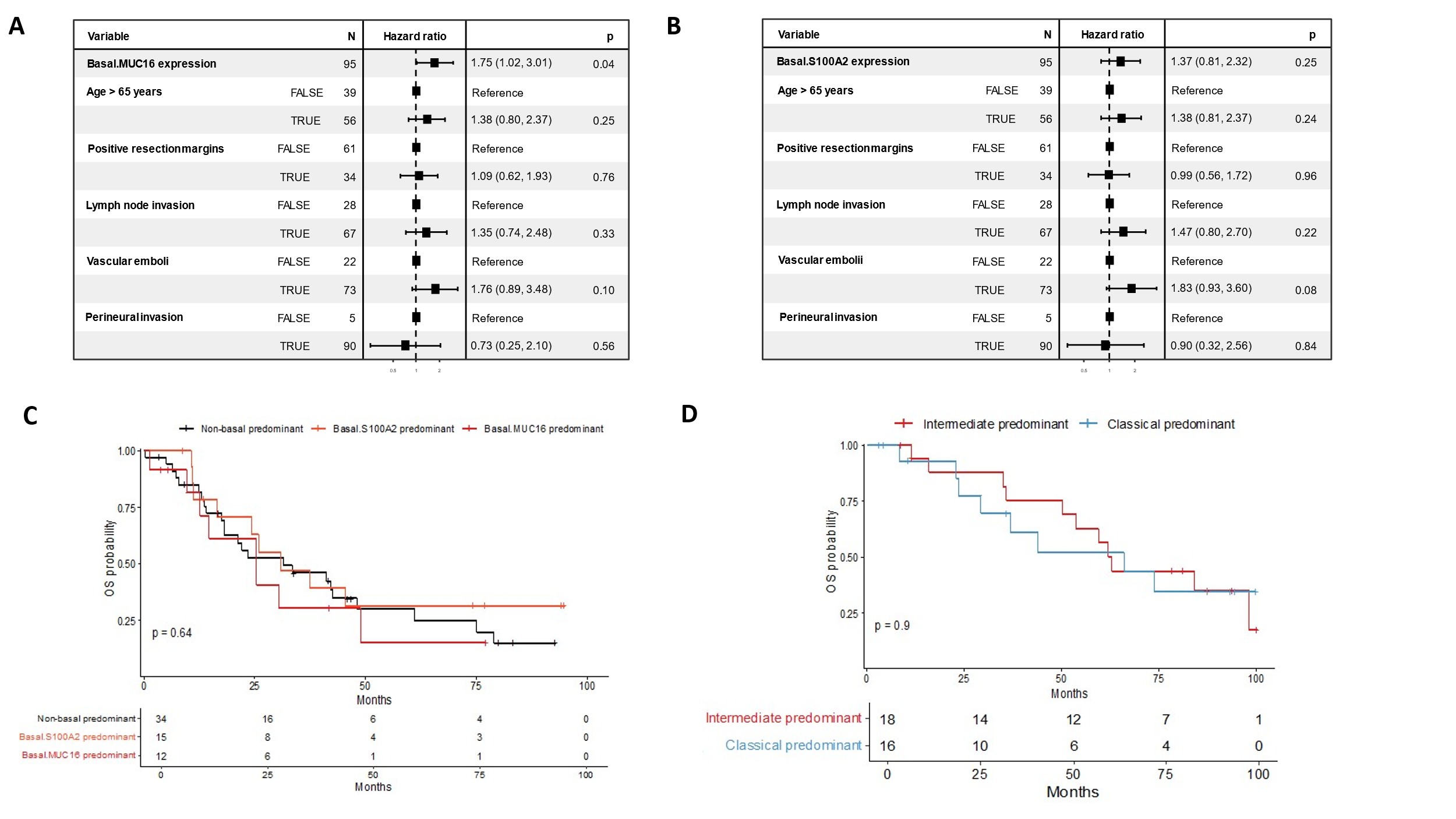


**Figure S12.** Survival according to cluster. Multivariate analysis for (A and B) the basal expression and clinicopathological factors regarding overall survival (*n* = 95 patients). Overall survival curves according to predominant cluster in (C) basal-expressing PDAC (*n* = 61 patients) and (D) basal-free PDAC.


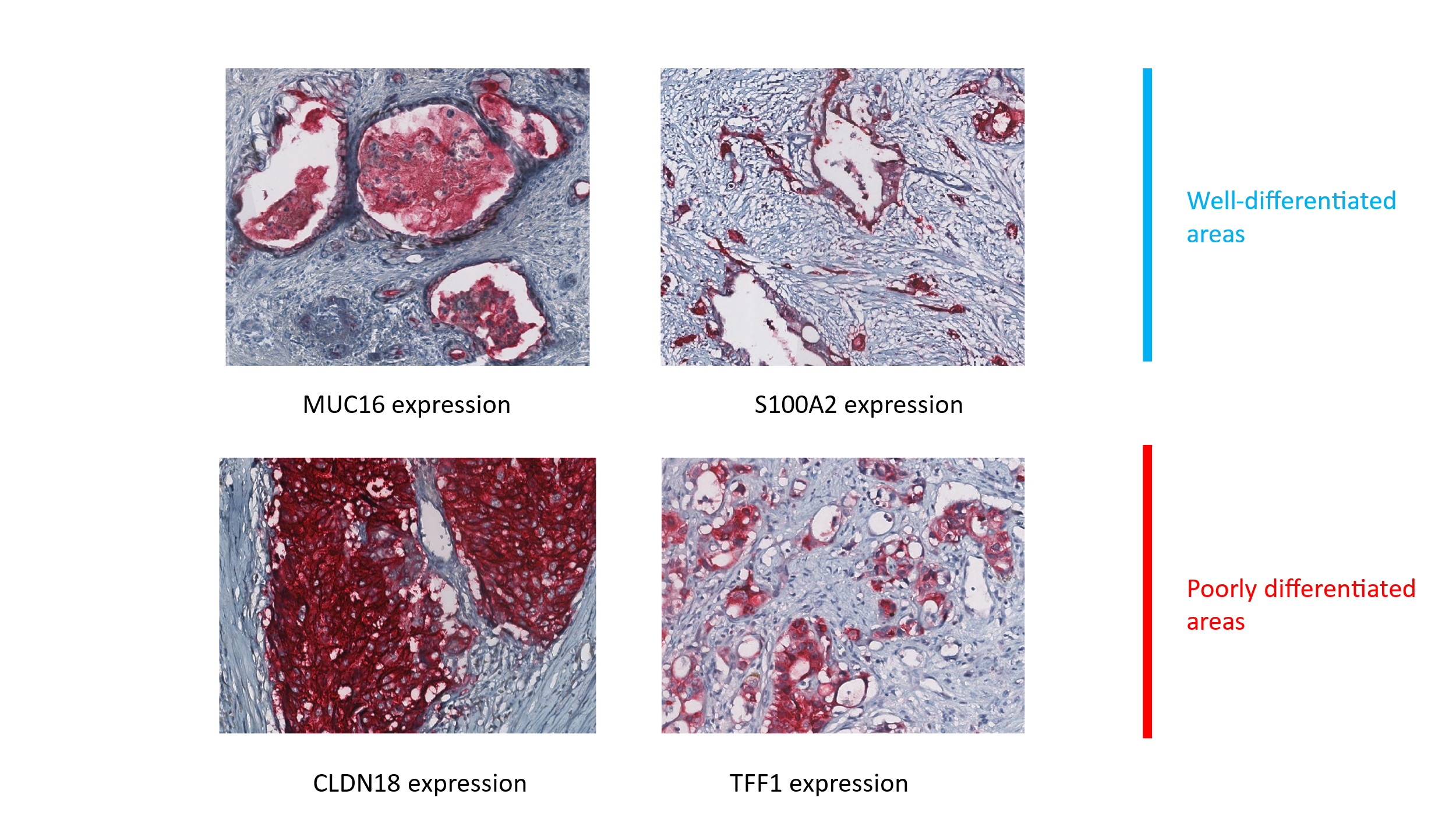


**Figure S13.** IHC expression of MUC16, CLDN18, S100A2, and TFF1 in poorly and well-differentiated areas.


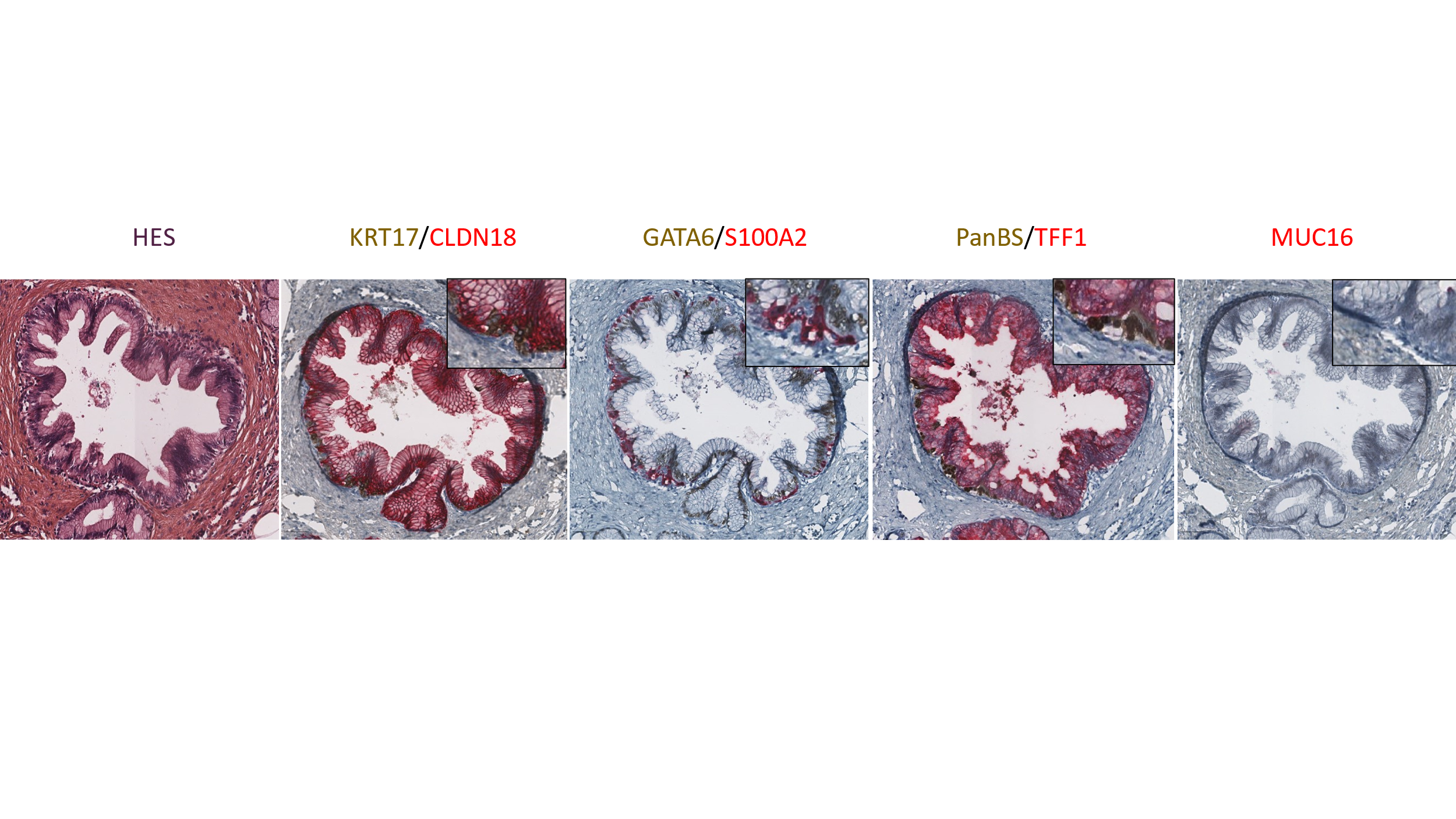
**Figure S14.** Expression of IHC panel in pancreatic intraepithelial neoplasia. A zoomed inset is incorporated at the upper right of each IHC image.


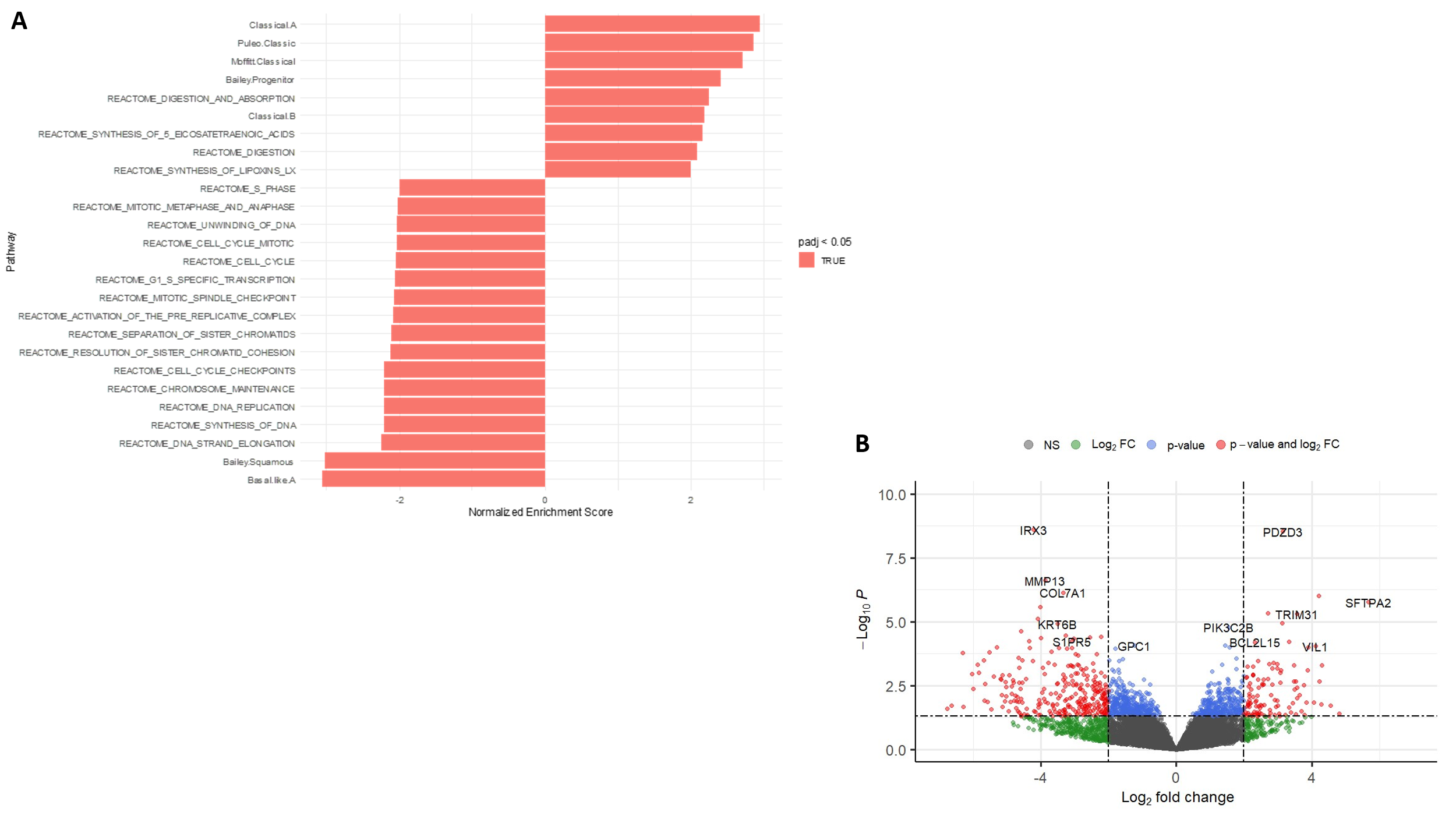


**Figure S15.** Differential analysis between basal clusters. (A) GSEA analysis showing signaling pathways that are differentially regulated and statistically significant (adjusted *p* value < 0.05) between Basal.MUC16 and Basal.S100A2 tumors. (B) Volcano plot showing differentially expressed genes between Basal.S100A2 and Basal.MUC16 tumors. Dashed lines indicate threshold of significant gene expression, defined as log2-transformed fold-change ≤ −2.0 and ≥ 2.0 with adjusted *p* value < 0.05.

**
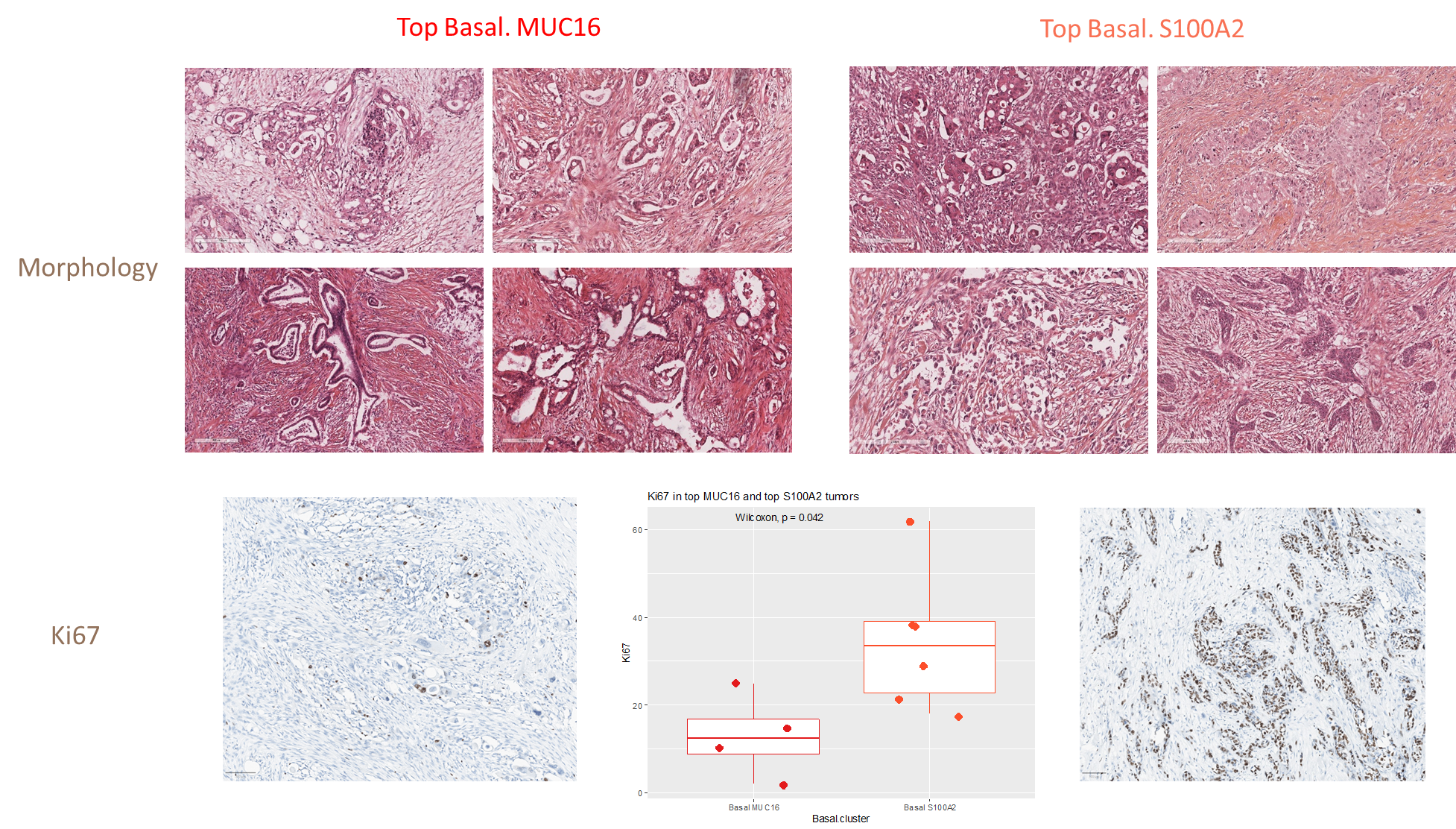
**

**Figure S16.** Morphology and proliferation comparison between basal clusters. Highly predominant Basal.MUC16 (*n* = 4) were more gland-forming/cribriform morphology, highly predominant S100A2 tumors (*n* = 6) were non-gland-forming/adenosquamous. The proliferation index in the tumor area (Qupath-based computer-assisted Ki-67 count) was higher in S100A2 tumors (*p* = 0.04).**Table S1.** Characteristics of the 24 tested antibodies.

| **Antibody to** | **Dilution** | **Clone** | **Species** | **Supplier** | **Marking location** |
| --- | --- | --- | --- | --- | --- |
| c-MET | 1/50 | D1C2 | Rabbit | Cell Signaling Technology, Danvers, MA, USA | Cytoplasmic |
| KRT6A - CK6A | 1/200 | polyclonal | Rabbit | Thermo Scientific, Waltham, MA, USA | Cytoplasmic |
| S100A2 | 1/1000 | EPR5392 | Rabbit | Abcam, Cambridge, UK | Cytoplasmic, nuclear |
| PanBasal | 1/100 | p63/CK5/CK14 | Mouse | Zytomed, Berlin, Germany | Cytoplasmic, membranous, nuclear |
| SNAI2 | 1/100 | polyclonal |  | Santa Cruz Biotechnology, Dallas, TX, USA | Nuclear |
| KRT17 - CK17 | 1/50 | E3 | Mouse | Thermo Scientific | Cytoplasmic |
| EGFR | 1/50 | EGFR 113 | Mouse | Novocastra, Leica microsystems, Nanterre, France | Membranous |
| SCL29A1- hENT1 | 1/200 | 10D7G2 | Mouse | Not commercially available [37] | Membranous, cytoplasmic, |
| SCL16A3 - MCT4 | 1/400 | polyclonal | Rabbit | Sigma-Merck, Darmstadt, Germany | Membranous |
| MUC16 | 1/250 | X325 |  | Abcam | Cytoplasmic |
| AGR2 | 1/1000 | D9V2F | Rabbit | Cell Signaling Technology | Nuclear |
| CYP24A1 | 1/50 | polyclonal | Rabbit | Biotechne, Minneapolis, MN, USA |  |
| HMGA2 | 1/25 | D1A7 | Rabbit | Cell Signaling Technology | Nuclear |
| GATA6 | 1/200 | D61E4 | Rabbit | Cell Signaling Technology | Nuclear |
| TFF1 | 1/100 | D2Y1J | Rabbit | Cell Signaling Technology | Cytoplasmic |
| CLDN18 | 1/50 | polyclonal | Rabbit | Sigma-Merck | Cytoplasmic, membranous |
| HNF4A | 1/100 | polyclonal |  | Sigma-Merck | Nuclear |
| REG4 | 1/100 | polyclonal | Rabbit | Thermo Scientific |  |
| HNF1A | 1/50 | polyclonal | Rabbit | Abcam | Nuclear |
| KRT20 - CK20 | 1/200 | K020.8 | Mouse | DAKO, Les Ulis, France | Membranous |
| CDX2 | 1/750 | EPR2764Y | Rabbit | Abcam | Nuclear |

**Table S2.** References chosen from literature review for *in silico* marker selection of basal and classical subtypes.

| **Title** | **Reference** |
| --- | --- |
| Transcription phenotypes of pancreatic cancer are driven by genomic events during tumor evolution | [3] |
| GATA6 expression distinguishes classical and basal-like subtypes in advanced pancreatic cancer | [20] |
| Subtypes of pancreatic ductal adenocarcinoma and their differing responses to therapy | [21] |
| Genomic analyses identify molecular subtypes of pancreatic cancer. | [22] |
| HNF1A recruits KDM6A to activate differentiated acinar cell programs that suppress pancreatic cancer | [23] |
| Virtual microdissection identifies distinct tumor- and stroma-specific subtypes of pancreatic ductal adenocarcinoma | [24] |
| Purity independent subtyping of tumors (PurIST), a clinically robust, single-sample classifier for tumor subtyping in pancreatic cancer | [25] |
| TP63-mediated enhancer reprogramming drives the squamous subtype of pancreatic ductal adenocarcinoma | [26] |
| Keratin 17 identifies the most lethal molecular subtype of pancreatic cancer | [27] |
| Isoforms of MUC16 activate oncogenic signaling through EGF receptors to enhance the progression of pancreatic cancer | [28] |

**Table S3.** Immunohistochemistry: basal expression according to tumor differentiation in Cohorts 2 and 3.

| **Cohort** | **Population** | ***n*** | ***n* (%)**  **Basal-expressing** | ***n* (%)**  **No basal expression** |
| --- | --- | --- | --- | --- |
| **Cohort 3 TMA-based** | Whole population | 148 | 113 (75.3%) | 37 (24.7%) |
|  | Well-differentiated tumors | 74 | 53 (71.6%) | 21 (28.4%) |
|  | Moderately differentiated tumors | 48 | 40 (83.3%) | 8 (16.7%) |
|  | Poorly differentiated tumors | 26 | 19 (73.1%) | 7 (26.9%) |
| **Cohort 2 Whole-slide-based** | Whole population | 95 | 61 (64.2%) | 34 (35.8%) |
|  | Well-differentiated tumors | 36 | 17 (47.2%) | 19 (52.8%) |
|  | Moderately differentiated tumors | 45 | 33 (73.4%) | 12 (26.6%) |
|  | Poorly differentiated tumors | 14 | 11 (78.6%) | 3 (21.4%) |
